# Exploring the Interface of Hyperconjugation and Polarizability—Enhancing Pedagogies for Charge Stabilization in Organic Chemistry

**DOI:** 10.1021/acs.jchemed.6c00708

**Published:** 2026-07-28

**Authors:** Mark C. Elliott, Kasimir P. Gregory, Colan E. Hughes, Benjamin D. Ward

**Affiliations:** † 2112 School of Chemistry, Cardiff University, Park Place, Cardiff CF10 3AT, U.K.; ‡ School of Science and Technology, University of New England, Armidale, NSW 2351, Australia

**Keywords:** First-Year Undergraduate/General, Organic Chemistry, Physical Chemistry, Misconceptions/Discrepant Events, Alcohols, Alkanes, Ammonium Compounds, Carbocations, Covalent Bonding, Computational Chemistry

## Abstract

The stabilization of positive and negative charges by alkyl groups (in particular) in organic compounds is often described as polarizability, but it can also be described as hyperconjugation (and related stereoelectronic effects). To a large extent, how one describes charge stabilization depends on the bonding model used. One can only talk about hyperconjugation in the context of a localized bonding model (hybridization). However, using this bonding model, it is less intuitive to talk about polarization of the whole molecule, which requires full molecular orbitals. It is important in chemical education to be aware of the limitations and intersections of the models we are using. In this work, we show that the polarization of alkyl chains in representative molecules, either by a computationally applied field or by a charge in the molecule, is reflected in changes in hyperconjugative interactions throughout the structure in a logical and predictable manner. This makes a clear and accessible point that hyperconjugation and polarizability are simply two models (at least for charge stabilization) that explain the same phenomenon. In this work, we identify advantages and limitations of each explanation, giving educators examples that can be used in teaching at various levels.

## Graphical abstract

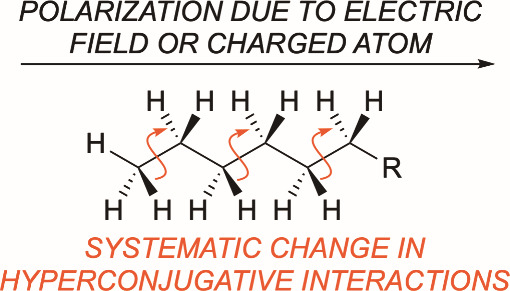

## Introduction

When we teach the stabilization of charge (e.g., carbocations) in nonconjugated organic molecules, the usual explanations involve one or more of inductive,^1^ hyperconjugation^2^ and polarizability^3^ effects. We have recently demonstrated that the inductive effect of alkyl groups in neutral molecules is electron-withdrawing.^4^ This has implications for the textbook teaching of aspects such as carbocation stabilization and amine basicity, for which an inductive effect is often invoked. Furthermore, we have shown that the inductive effect as presented in textbooks needs refinement, since the ability of an electronegative element to stabilize negative charge is defined primarily by its polarizability rather than by its electronegativity.^5^ In this work, we will highlight the fact that (at least in the context of charge stabilization) hyperconjugation and polarizability are two ways of presenting the same effect. Hyperconjugation is generally the more accessible pedagogy, since it readily lends itself to clear diagrammatic representation. However, there are situations in which the general polarizability of alkyl groups provides more insight. Although carbocations are a significant focus of the work, we will discuss the stabilization of alkoxide and ammonium ions, to which some of the same problems apply. We will present clear and accessible examples using modern computational methods to enhance the teaching of structural organic chemistry. The organization of the manuscript is as follows:

1. A brief discussion of the current textbook situation, focusing on carbocation stabilization.
2. A survey of the equivalence of hyperconjugation and polarizability, based on representative literature examples and a discussion of the nature of such orbital effects.
3. A series of examples, in which we explore the polarization of molecules by electric fields or charges, and see systematic changes in hyperconjugation interactions.
4. Recommendations for changes to teaching as a result of this work.

## Discussion of Current Pedagogies for Charge Stabilization

For carbocation stabilization, textbooks^6–9^ and online resources^10^ list all three effects (inductive, hyperconjugation, polarizability), sometimes with comments on the relative importance of each.

The inductive effect of alkyl groups in neutral molecules has recently been shown to be electron-withdrawing relative to hydrogen,^4,11,12^ with no significant difference between alkyl groups.^13^

Where the inductive effect is given in some textbooks^6,8,14^ to explain carbocation stabilization, the logic is generally that a carbocation carbon is more electronegative than a methyl group carbon such that we would see polarization of a C–C bond as shown in Figure 1. This is, of course, true. However, since hydrogen is less electronegative than a methyl group carbon, the carbocation carbon should be *even more* inductively electron-withdrawing from hydrogen. The inductive effect of alkyl groups, solely due to the differing electronegativity between the carbocation and alkyl carbons, cannot explain the carbocation stabilization.

**Figure 1:**
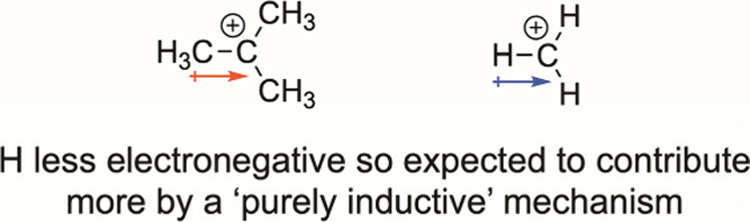
Methyl groups should *destabilize* carbocations by a “purely inductive” mechanism.

This leaves hyperconjugation^6,7,15–23^ and polarizability^24,25^ as textbook explanations for the stabilization of carbocations by alkyl groups. The fact that there are comments in textbooks about the relative importance of each would lead a student to conclude that they are distinct effects.

Largely, the current situation has arisen for historical reasons. Hyperconjugation has its origins in the Baker-Nathan effect,^26^ which fell out of favor in the 1960s when it was found that the original observations of Nathan and Baker were better explained as solvent effects.^27^ In Morrison and Boyd’s classic textbook,^28^ the term ‘inductive effect’ was used for carbocation stabilization, but the explanation is consistent with polarizability. Karty explains this effect in terms of the methyl group carbon withdrawing electron-density from its hydrogen atoms and passing them on to the carbocation, with a diagram similar to Figure 2 used,^25^ although this diagram is not present in later editions of this textbook. Again, this describes polarizability although a student could confuse this with an inductive effect. One key pedagogical disadvantage of polarizability is that there seems to be no clear unambiguous diagrammatic representation.

**Figure 2:**
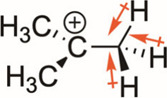
Potential representation of methyl group polarizability that is not sufficiently distinct from an inductive effect.

More recently, hyperconjugation^29^ has become the predominant explanation for carbocation stabilization by alkyl groups. In earlier textbooks, resonance canonical forms were drawn with only brief mention of molecular orbitals.^21^ The molecular orbital explanation now dominates, and hyperconjugation is generally drawn using localized hybrid C–H bond orbitals, with the importance of proper alignment of the C–H bond with the empty p-orbital generally stated, as shown in Figure 3 for the *t*-butyl carbocation.

**Figure 3:**
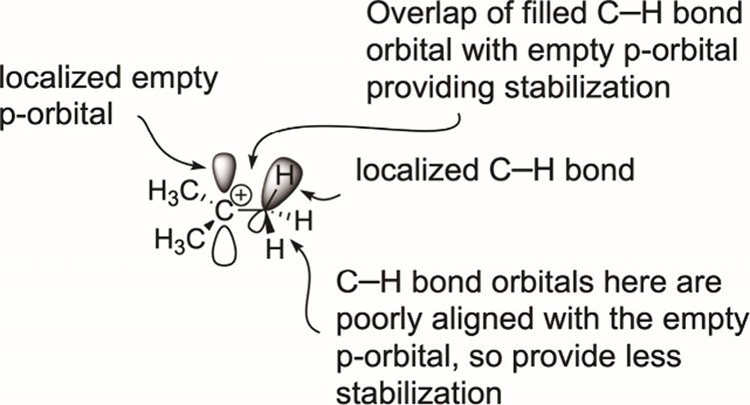
Typical textbook representation of hyperconjugation.

In most introductory organic chemistry textbooks, carbocation stabilization by alkyl groups is presented only in terms of methyl groups. This is appropriate, since the main focus is to develop an understanding of the fact that alkyl groups stabilize positive charge, and that more alkyl groups provide more stabilization. It is rare for there to be any consideration of the effects of different-sized alkyl groups. In older textbooks, the presentation of hyperconjugation is limited to C–H bonds, and in some older textbooks it is explicitly stated that this is the case.^30,31^ In fact, larger alkyl groups generally provide more stabilization. To explain this using hyperconjugation of ‘immediate’ bonds we would have to consider the relative ability of C–H and C–C bonds to participate in hyperconjugation.^17^ This, in turn, depends on proper alignment of the various bonds with the carbocation p-orbital. By appreciating the intersection of hyperconjugation and polarizability, many of these problems disappear.

## How Are Hyperconjugation and Polarizability Related?

To answer this question, we must consider how molecules (and their orbitals) can be polarized, and what this means from a localized bonding perspective. Whenever we discuss the stabilization of charge in a molecule, we are effectively considering a full localized charge on an atom, and asking what the substituents (alkyl groups, for the purpose of this discussion) do relative to a suitable reference point (usually hydrogen).

Molecular orbitals, also referred to as ‘canonical’ molecular orbitals, span entire molecules, although some orbitals are more localized on one part of a molecule than another. These orbitals can be dissected, using tools such as NBO,^32^ into localized hybrid orbitals.^33^ If we do this for the *t*-butyl carbocation we obtain three localized C–C bonds, nine localized C–H bonds and a p-orbital that represents the carbocation (Figure 3). In the absence of hyperconjugation, each C–H bond would have an occupancy of 2.00 e, and the p-orbital would be empty. Hyperconjugation is the interaction of the C–H bond orbital with the empty p-orbital, and results in sharing of electron-density. Because of this sharing, the average occupancy of the C–H bond orbitals is 1.91 e, with the p-orbital having 0.31 e. Each methyl group in the t-butyl carbocation has one C–H bond that is well-aligned with the empty p-orbital, and two that are poorly aligned (Figure 3), with the aligned bond providing more stabilization. In the language of NBO calculations, the interaction between the C–H bonds and the carbocation is a second-order perturbation, and it represents interactions between the localized orbitals. Since these interactions were already present in the canonical molecular orbitals, it is inevitable that we will see this polarization reflected in the NBO calculations for hyperconjugation and related stereoelectronic effects. We will provide clear examples showing this to be the case.

In essence, canonical molecular orbitals are polarized by charge, and *hyperconjugation is what this polarization looks like when viewed through the lens of localized bonding*. Although not present in our textbooks or online teaching resources, the link between hyperconjugation and polarizability is present in the research literature. Weinhold and Landis note that the NBO program^34^ “offers useful tools for ... dissecting molecular dipole moment or polarizability into localized bond dipole and resonance-type contributions of recognizable chemical origin.”^35^ Alabugin notes the importance of polarizability in controlling the magnitude of hyperconjugation effects.^36^ The classic physical organic chemistry literature also provides evidence of the equivalence of hyperconjugation and polarizability. Hyperconjugation parameters introduced by Kreevoy and Taft^37^ were considered unnecessary by Charton and Charton,^38^ since they felt that electrical, steric and polarizability effects were sufficient. Roberts and Moreland used bicyclo[2.2.2]­octanecarboxylic acids to define substituent inductive effects on the basis that conjugation effects would not apply.^39^ However, it is clear that substituent effects in such systems can be transmitted through the bicyclooctane ring system.^40^ These compounds are prototypical systems for ‘double hyperconjugation’,^41–44^ in which a carbocation is perceived to be stabilized by localized orbital interactions represented by resonance forms such as the following (Figure 4). The link between polarizability and double hyperconjugation has already been made.^45^

**Figure 4:**
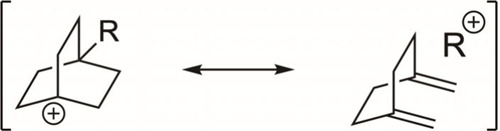
Resonance canonical forms for double hyperconjugation.

Aside from carbocation chemistry, larger alkyl groups better stabilize negative charge in alkoxide^46^ anions in the gas phase. This stabilization was attributed to polarizability, and is covered as such in textbooks.^47^ Jones and Fleming^48^ describe this effect as “electron delocalization into alkyl group antibonding orbitals” (i.e., negative hyperconjugation, as it has been described in the research literature^49^). Larger alkyl groups similarly stabilize amine^50^ anions and ammonium cations^51^ in the gas phase. Schubert, Murphy and Robins invoked alkyl group polarizability to explain the ability of alkyl groups to stabilize negative charge in reactions.^52^

In this work, we will show that the hyperconjugation explanation, as is generally presented in textbooks, does not capture all aspects of charge stabilization. Conversely, a general polarization effect is perhaps more nebulous. By demonstrating how hyperconjugation relates to polarizability, the two approaches complement one another. The computational study which underpins this work allows us to present a more holistic pedagogy of polarization and hyperconjugation. We will present our results and then discuss the implications for the teaching of these areas in undergraduate organic chemistry courses.

## A Note on Terminology

There are various stereoelectronic effects that might fall under the general umbrella term of ‘hyperconjugation’ but that can be defined separately as shown in Figure 5.^53^ There are some additional, more esoteric, terms that we will use, such as homohyperconjugation and double hyperconjugation, which we will define later.

**Figure 5:**
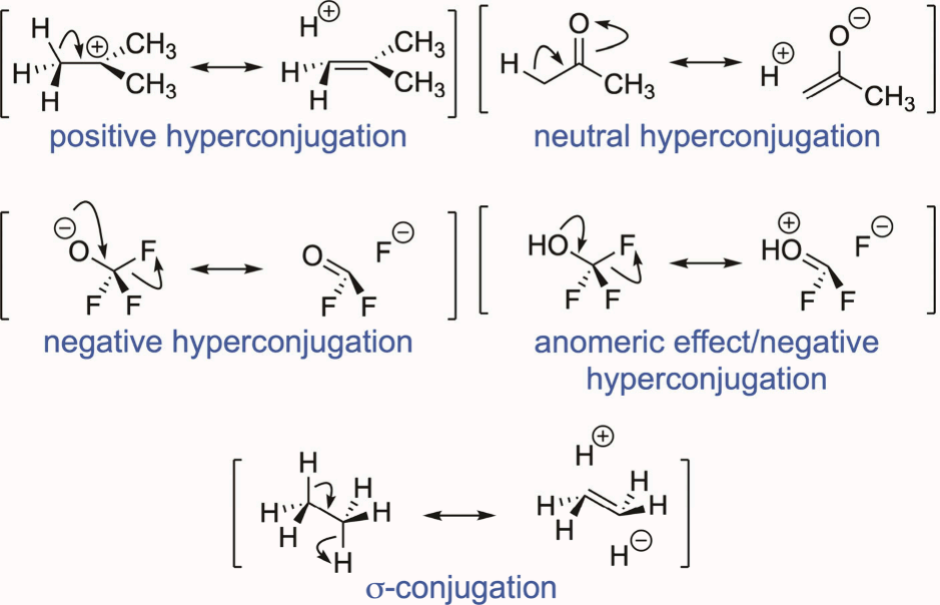
Classification of hyperconjugative stereoelectronic interactions.

Fundamentally, all these effects are the interactions between localized orbitals that have been partitioned from the canonical molecular orbitals, and as such we consider the distinction between them to be largely semantic (although useful in teaching). When discussing specific interactions, we will endeavor to use the correct specific terminology, but we will use ‘hyperconjugative interaction’ (as used by Alabugin to describe anomeric effects^54^), or simply ‘hyperconjugation’ when discussing the effects more generally.

## Results and Discussion

### Computational Methods

We have chosen to use NBO calculations in conjugation with charge decomposition analysis and calculations in which an electric field is applied to a molecule to address this issue. There are several methods for the partitioning of charge between atoms in a molecule.^55^ In this work, we will present CM5 charges,^56^ for reasons discussed in the Supporting Information. Other charge models (Hirshfeld,^57^ NPA,^58^ CHELPG,^59^ DDEC6 ^60^) lead to the same conclusions. Other charge models are discussed in the Supporting Information. NBO calculations^61^ use the NBO 3.1 module within Gaussian. MP2 calculations^62^ with the aug-cc-pVTZ flexible orbital basis set^63–65^ were used since this level of theory has been found to give good results for calculating dipole moments and polarizabilities.^66^ Calculations were carried out with Gaussian 09 software Revision D.01.^67^ All structures were fully optimized. Apart from the field calculations, frequency analysis was undertaken to confirm the presence of minima. Conformational analysis was not carried out, but similar conformers of each structure are presented to allow meaningful comparisons.

### Hexane

In the first instance we will avoid many of the potential complications by focusing on a neutral molecule, hexane, to demonstrate the equivalence of hyperconjugation (specifically σ-conjugation) and polarizability.

Polarizability^3^ is the response of a molecule to an electric field.^68^ This applied field can be simulated computationally, so we can polarize a molecule computationally and probe the effect of this on the charge distribution and molecular orbitals. The structure of hexane was optimized in the absence and presence of an electric field (0.005 au) along the axis of the molecule, inducing a dipole moment of 1.2 D. This is represented diagrammatically using ‘electrodes’, so that in Figure 6 the electron-density (as shown for the lowest energy bonding molecular orbital) in the molecule is shifted to the right. Similar effects are seen with all molecular orbitals leading to distortion of the overall electron-density. This is what we would expect.

**Figure 6:**
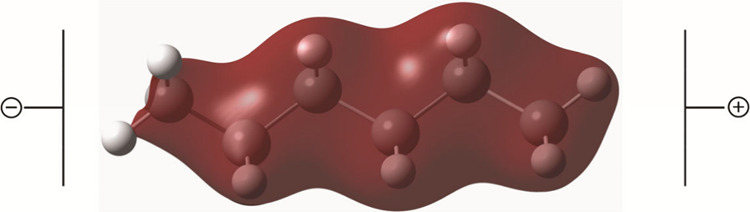
First bonding molecular orbital of hexane in electric field (0.005 au).

Since calculated atomic charges are partitioned from the overall electron density, polarization will lead to changes in these charges. The changes are small, as might be expected, but they are consistent (Figure 7). Carbon and hydrogen atoms on the right gain electron-density due to polarization, while those on the left lose electron-density. The largest redistribution of charge is at the termini of the molecule by about an order of magnitude. This is in line with work showing that the interior atoms in molecules have considerably reduced polarizability.^69^ It is also consistent with Figure 6.

**Figure 7:**
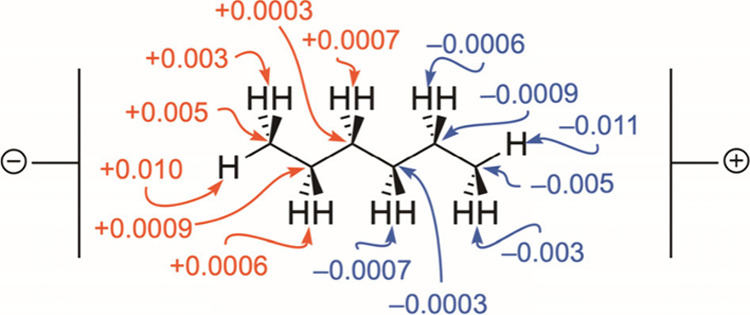
Changes in CM5 calculated atomic charges (e) on carbon and hydrogen in hexane as a result of applied field (0.005 au).

We can then use NBO calculations to determine the relevant interactions from the second-order perturbation matrix. We will focus on σ-conjugation between a single pair of antiperiplanar vicinal C–H bonds. With the molecule polarized in the direction shown, the C–H σ to C–H σ* interaction from a to b increases, while the interaction from b to a is decreased (Figure 8). The same is true for all other σ-conjugation interactions (full data in Supporting Information).

**Figure 8:**
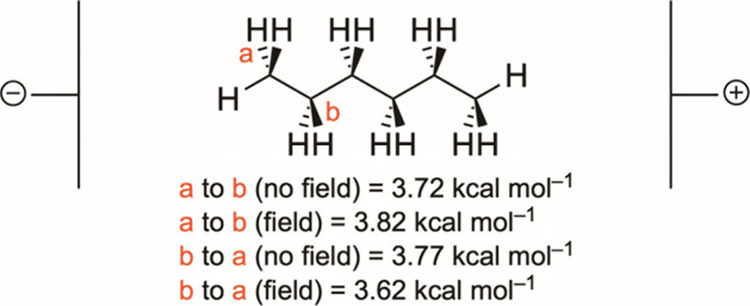
Selected C–H σ to C–H σ* NBO interactions in hexane.

This is therefore a simple system in which the molecule can be (computationally) polarized, and the consequences are reflected in systematic changes to the hyperconjugative interactions. This idea, while simple, has significant implications for the discussion of charge stabilization in organic chemistry textbooks. **Specifically, one should not discuss stabilization by hyperconjugative interactions (such as σ-conjugation) and polarizability in a way that implies they are separate effects**. To reiterate an earlier point, polarization/polarizability is the response of the molecular orbitals to charge/electric field and (changes in) hyperconjugation is what this looks like from a localized bond perspective.

### 1-Fluoropentane

With fluoropentane, we have several added aspects that show the benefit of one or another approach. The unsymmetrical molecule can be polarized along the length of the carbon chain in either direction. We have previously used this method to demonstrate how partial atomic charge changes upon polarization,^5^ and we now extend this work to show that the σ-conjugation interactions change in line with the polarization. Calculated fields of 0.005 au and 0.02 au were used, the latter to enhance the effects, with the geometry optimized in each case. There is existing polarization from the electronegative fluorine that we either enhance or oppose.

Calculated charges for 1-fluoropentane are shown in Figure 9 (gray line), with charges for CH_3_ and CH_2_ groups combined into a single data point. We can then see the impact of a field of +0.02 au (defining a positive field as polarizing electron-density toward F) in the red line. In line with the calculations on hexane, the impact is felt most strongly at the extremities of the molecule. There are small net changes in charge at the intervening atoms—these atoms both accept and donate electron-density. With the field in the opposite direction (−0.02 au; dark blue line) electron density is notionally being transferred from CH_2_F to the CH_3_ group. With fields of 0.005 au in either direction (orange, light blue lines) the effects are similar but smaller. The extent of charge transfer is not identical with the field applied in different directions. This is to be expected, since there is existing polarization of the C–F bond and applying the field in this direction is enhancing an existing bias, whereas applying the field in the opposite direction must overcome this bias.

**Figure 9:**
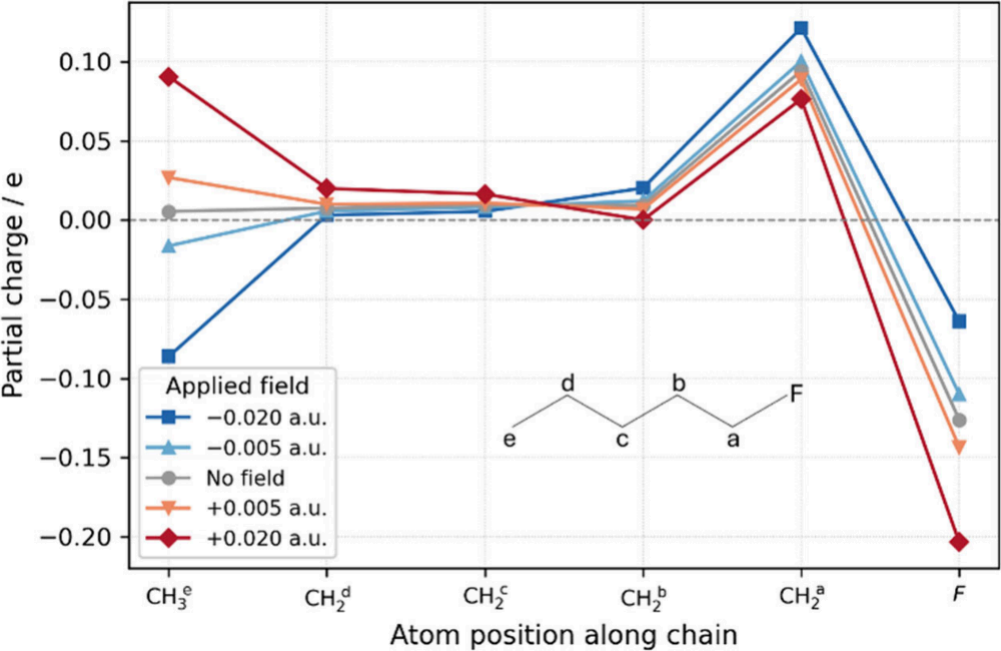
Amalgamated CM5 charges (e) for 1-fluoropentane. A positive field is designated as polarizing electron-density toward F.

As before, we will focus on a single σ-conjugation NBO interaction. The C–C σ to C–F σ* interaction from a to b increases with the field in the direction shown, while the corresponding interaction from b to a is decreased (Figure 10). The same is true for all other σ-conjugation interactions. Naturally, with the field in the opposite direction, the changes are in the opposite direction. Full NBO data are given in the Supporting Information.

**Figure 10:**
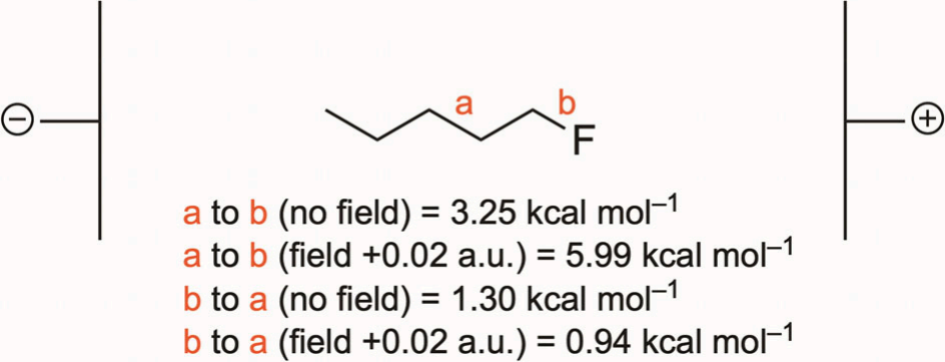
Selected NBO interactions (kcal mol–1) in 1-fluoropentane as a result of computationally applied field (+0.02 au).

Again, then, we have an effect induced by polarization of the molecule, and we see the consequences of this as changes in the σ-hyperconjugative interactions. There are potential advantages to considering the specific stereoelectronic effect. Selected bond lengths are shown in Table 1. With the applied field in the positive direction, we find that C1–F is lengthened as the fluorine becomes more negative. With the stronger field, the C3–C2 bond is also lengthened. Both changes can be rationalized by resonance curly arrows (a). The C2–C1 bond is shortened, which is consistent with this analysis. With the applied field in the negative direction, the C1–F bond is shortened, and C2–C1 lengthened, corresponding to resonance form (b). The corresponding NBO interaction (fluorine lone-pair to C1–C2 σ*) increases by 0.78 kcal mol^–1^ with a field of –0.02 au These changes are entirely in line with a fluorine anomeric/negative hyperconjugation effect.^70^

**Table 1:**
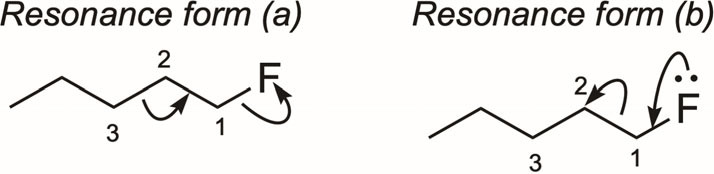
Calculated Bond Lengths (Å) in 1-Fluoropentane with Applied Field (a.u.)

Field (a.u.)	C3–C2	C2–C1	C1–F
–0.02	1.524	1.520	1.370
–0.005	1.524	1.511	1.389
no field	1.524	1.509	1.396
+0.005	1.525	1.507	1.404
+0.02	1.535	1.502	1.441

This shows a distinct advantage of using the hyperconjugative bonding approach. However, we recommend avoidance of expressions such as ‘**these changes in bond length are because of hyperconjugation**’ in favor of ‘**these changes in bond length can be explained using the hyperconjugation bonding model**’. In this, we include the σ-conjugation and the anomeric/negative hyperconjugation shown above.

### Pentanol and Pentoxide

Pentanol is slightly more acidic than ethanol in the gas phase,^71^ and this can easily be reproduced computationally (see Supporting Information). Polarizability^46^ and negative hyperconjugation^49^ have both been used at various times to explain the relative stability of alkoxide anions. The typical negative hyperconjugation interaction in pentoxide, shown at the top of Figure 11, would lead a student to conclude that this is a short-range interaction. However, a second interaction (which might be called negative homohyperconjugation; bottom of Figure 11) is also present. Quite simply, we are seeing the fact that the negative charge is not localized on oxygen, and the NBO interactions are the representation of this delocalization in terms of localized orbitals. This is reflected in the charge redistribution in pentoxide compared to pentanol (Figure 12).

**Figure 11:**
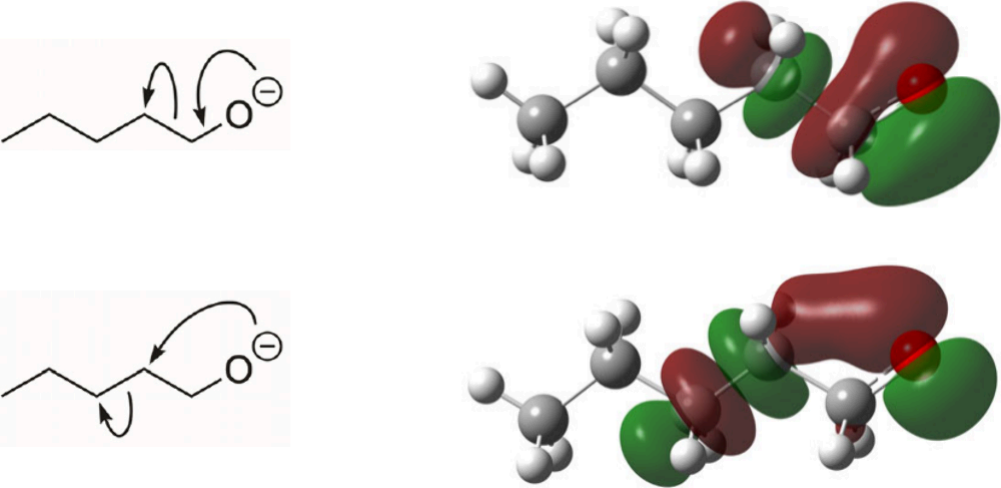
Hyperconjugative NBO interaction between one of the oxygen lone pairs and the C1–C2 (top) and C2–C3 (bottom) σ* orbitals.

**Figure 12:**
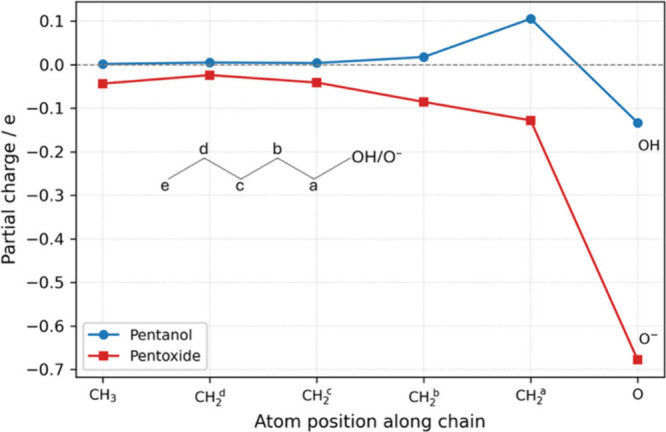
Calculated CM5 charges for pentanol and pentoxide.

The effect at C1 can be considered to be inductive, since an alkoxide oxygen is less electronegative than an alcohol oxygen.^72^ Donation to C2 and C3 can be rationalized by the resonance forms shown above, and changes at C4 and C5 can also be seen in the σ-conjugation interactions (Figure 13). However, it might be simpler to consider the overall effect as polarizability while recognizing that it can also be seen within specific σ-conjugation interactions.

**Figure 13:**
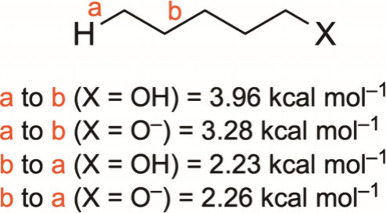
Selected σ-conjugation NBO interactions for pentanol and pentoxide.

### Pentylamine and Pentylammonium

Our final example before returning to carbocations is one that we would not generally use hyperconjugation for. Amine basicity is generally covered in the research literature as polarizability,^51^ although it is taught in some regions (UK A-Levels) as being due to the ‘increased availability of a nitrogen lone pair due to the positive inductive effect of alkyl groups’. We know this explanation is wrong, since we have shown^4^ that alkyl groups are inductively electron-withdrawing in neutral molecules.^11,12^

An ammonium group (electronegativity 4.3)^72^ is more electronegative than fluorine, but the positive charge has a much larger effect. The charge distribution in pentylammonium and in pentylamine is shown in Figure 14. The calculated CM5 charge on the pentylammonium NH_3_^+^ group is +0.58 e and the charge on the pentyl group is +0.42. The donation of electron-density from the pentyl group comes from all atoms, although the majority comes from CH_2_^a^. However, CH_3_^e^ donates more electron-density than CH_2_^c^ or CH_2_^d^, which is in line with the calculations shown above for 1-fluoropentane.

**Figure 14:**
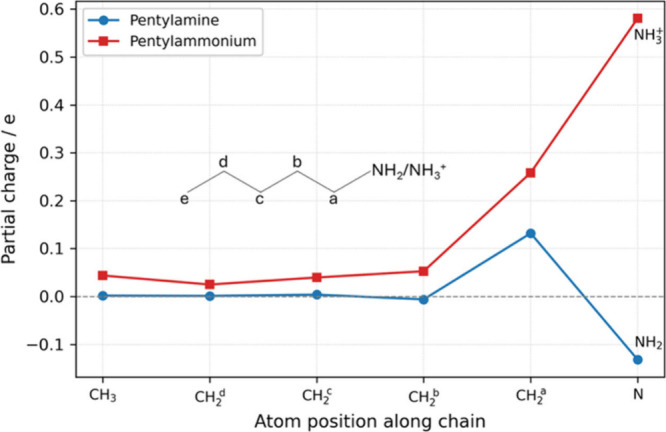
Calculated CM5 charges for pentylamine and in the pentylammonium cation.

As before, we see that σ-conjugation NBO interactions directed toward nitrogen are increased in ammonium compared to amine (e.g., bond a (σ) with bond b (σ*), Figure 15) while those directed away from nitrogen decrease. The same is true for other NBO interactions as given in the Supporting Information.

**Figure 15:**
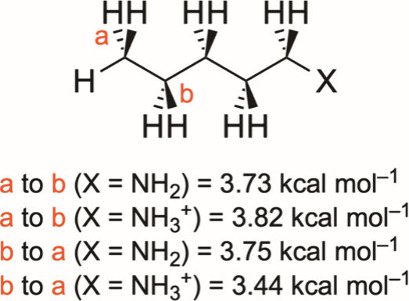
Selected NBO interactions in pentylamine and in the pentylammonium cation.

We therefore see that in a charged structure with no obvious acceptor orbital, the consequences of polarization are still seen in the NBO analysis.

### *t*-Butyl and 2-Methylhex-2-yl Carbocations

In most organic chemistry textbooks, carbocation stabilization by alkyl groups is presented only in terms of methyl groups, and we have already noted that in some older textbooks, hyperconjugation is explicitly stated to be a phenomenon involving C–H bonds.^30,31^ On this basis, a student might expect the *t*-butyl carbocation to be more stable than the 2-methylhex-2-yl carbocation (Figure 16), whereas according to calculated hydride ion affinity^73^ the latter is 4.1 kcal mol^–1^ more stable.

**Figure 16:**
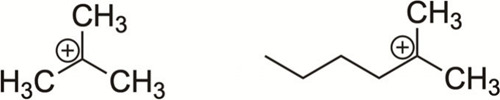
Structures of the t-butyl and 2-methylhex-2-yl carbocations.

In some textbooks, the ability of C–C bonds to participate in hyperconjugation is mentioned,^17^ but the focus is still on the ability of bonds directly adjacent to the carbocation exhibiting a specific orbital effect. The computational data show that the stabilization is provided by the entire butyl chain. Charge decomposition analysis (CM5), shows the carbocation carbon in the *t*-butyl cation having +0.21 e, compared to +0.20 e in the 2-methylhex-2-yl cation, which (although small) is consistent with the above energy differences. When comparing the carbocations to the corresponding alkanes (isobutane and 2-methylhexane) we find that each methyl group in the *t*-butyl carbocation loses 0.26 e, whereas each methyl group in the 2-methyhex-2-yl carbocation only loses 0.24 e. The butyl group in the 2-methylhex-2-yl carbocation loses 0.30 e. We might say that when the butyl group is present to donate more electron-density, each methyl group works ‘less hard’. Looking at the charges along the butyl chain (Figure 17), we find that only 0.17 e of the 0.30 e comes from the first CH_2_ group. The effect might be considered a general polarization effect, which can also be viewed as σ-conjugation. There is more donation from the terminal CH_3_ group than from the next two CH_2_ groups, exactly as we saw in the pentylammonium cation above.

**Figure 17:**
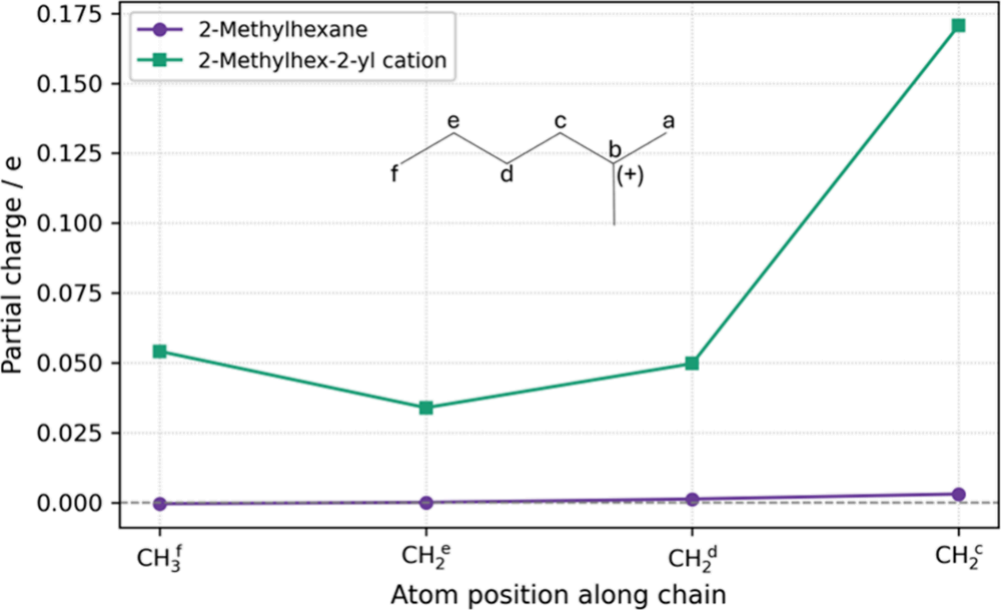
Calculated CM5 charges in the butyl group in the 2-methylhex-2-yl carbocation and in 2-methylhexane.

Consideration of the hyperconjugation interactions shows the complexity of the situation. On average, each CH_3_ group in the *t*-butyl carbocation ‘provides’ 41.5 kcal mol^–1^ of stabilization by C–H hyperconjugation. This corresponds to a large stabilizing interaction from the C–H bond that is aligned with the carbocation p-orbital, and smaller interactions from the C–H bonds that are less-well aligned (Figure 18). In the 2-methylhex-2-yl carbocation, the C3–C4 bond is perpendicular to the carbocation p-orbital (Figure 18) and there is calculated to be no hyperconjugation (<0.5 kcal mol^–1^). The two C–H bonds of the butyl substituent provide a total of 44.7 kcal mol^–1^ of stabilization, which is more than provided by three C–H bonds of a methyl group. In the 2-methylhex-2-yl carbocation, each methyl group provides an average of 36.0 kcal mol^–1^ of stabilization, which is lower that for the methyl groups in the *t*-butyl carbocation. This is consistent with the charge analysis above.

**Figure 18:**
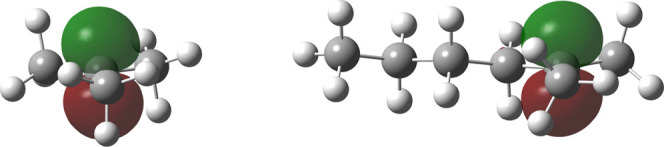
3D representations of the optimized structures of the t-butyl and 2-methylhex-2-yl carbocations, showing the carbocation ‘empty’ p-orbital (from NBO calculation).

If we simply accept that a butyl group is more polarizable than a methyl group,^74^ the greater stabilization of charge by a butyl group requires little consideration. We can also rationalize this as hyperconjugation if we note that the CH_2_ group receives electron-density from further down the chain (Figure 19) which it is able to pass on.

**Figure 19:**
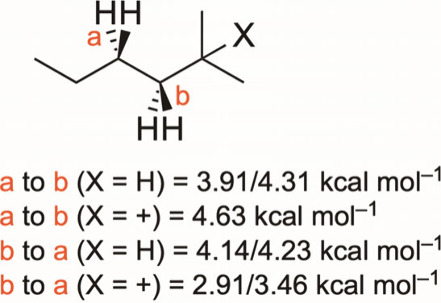
Selected σ-conjugation NBO interactions in the 2-methylhex-2-yl carbocation.

We see that the ‘immediate’ hyperconjugation is not a complete explanation for the stabilization of carbocations, but can be refined by the broader consideration of polarizability, which can also be seen as σ-conjugation interactions along the chain.

## Enhancing Pedagogies for Carbocation Stabilization

Carbocation stabilization is not always consistently presented in textbooks, and the link between hyperconjugation and polarizability is not present. By highlighting this link, we hope to streamline the teaching of charge stabilization, and to give educators specific examples that they can teach. We note that the calculations herein are not exceptionally difficult. It is relatively straightforward to calculate relative energies and charge distributions in small molecules that are well-suited to teaching.

### The Inductive Effect

There is a subtlety that we have deliberately avoided up to this point. According to the strict definition, based on experimentally observable effects,^1^ any effect due to hyperconjugation or polarizability is included within the definition of the inductive effect. Strictly speaking, carbocation stabilization by an alkyl group *is* an inductive effect but only because these two effects are directly included. However, the subtlety of this argument is likely to cause confusion to students of the subject. **We recommend that the term ‘inductive effect’ not be used in the context of carbocation stabilization by alkyl groups**. Alkyl groups are –I +R groups,^11,12^ and there is evidence from ^13^C NMR chemical shifts that the –I effect still operates in carbocations.^75^

### Advantages and Disadvantages of Hyperconjugation as a Pedagogy

The key advantage of hyperconjugation as a pedagogy is the accessible nature of diagrammatic representations (Figure 3; Figure 5). The limitation is that these diagrams imply that stabilization of carbocations by hyperconjugation is a short-range effect limited to bonds to atoms adjacent to the carbocation. We have to invoke ideas such as double-hyperconjugation (Figure 4) to consider longer range interactions, and these are rather artificial constructs. By focusing on hyperconjugation primarily from the perspective of methyl groups, most textbooks manage to conveniently avoid the issue. This is probably a good idea. For more advanced study, a broader selection of carbocations, including biologically relevant systems, can be presented. Where larger alkyl groups are included as examples, **it should be explicitly stated that larger alkyl groups provide more stabilization**. At this point, the link between polarizability and hyperconjugation can be made. A brief comment that **hyperconjugation is a localized bond model for a broader orbitalpolarization** is sufficient. In particular, **we would avoid discussing the relative contribution of C–H and C–C bonds to hyperconjugation**. As we have seen, in the 2-methylhex-2-yl carbocation the butyl group is able to provide more stabilization than the methyl group despite one bond (C–C) being perpendicular to the carbocation p-orbital. We should avoid giving the impression that we have two distinct effects: **(1) a short-range*specific orbital overlap effect*(hyperconjugation) and (2) a separate*general longer-range polarizability effect***. Rather than asking whether stabilization is due to hyperconjugation or to polarizability, we might better ask which model provides the best insight.

### Presenting Polarizability as a Pedagogy?

The key problem with polarizability as a pedagogy is the lack of an unambiguous visual representation. One can draw arrows on bonds to show the direction of polarization (Figure 2), but this can easily be confused with an inductive effect. Although Fleming and Jones describe alkoxide stabilization as an orbital effect, the diagram used is conceptually similar to Figure 2.^48^ A different approach is used by Loudon and Parise^47^ for alkoxide anion stabilization, in which a schematic representation (like Figure 20) of the electron cloud of alkyl groups is shown.

**Figure 20:**
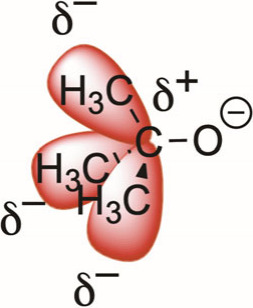
Diagram indicating methyl group polarizability in the *t*-butoxide anion (after Loudon and Parise^47^).

There is no unique solution to this problem. On balance, we recommend a ‘simple’ explanation that it is the molecular orbitals of the charged species that are being polarized, and hyperconjugation focuses on the contribution of individual bonds, which is only part of the story. Depending on the level of study, examples from this work would serve well to explain how charge is donated from (or accepted by) an entire alkyl chain rather than by individual bonds proximal to the charge.

### Hyperconjugation—The Need for Bond Alignment

In a simple carbocation such as *t*-butyl, the barrier to C–C bond rotation is low such that each C–H bond will spend some time aligned with the empty p-orbital, and the bonds that are not well-aligned still provide some stabilization. Consider Figure 21, in which the carbocation orbital, denoted as the p_*z*_ orbital, is eclipsed with bond a. According to a localized bond model this would provide the largest amount of hyperconjugation. The methyl carbon is sp^3^ hybridized, and according to the axes chosen, bond a is derived from the s-orbital and the p_*z*_ and p_*y*_ orbitals. As we rotate the CH_3_ group about the *y*-axis, the balance of p_*x*_ and p_*z*_ contribution will vary between bonds a, b and c, but (to a first approximation) the total hyperconjugation interaction will be the same. **Although bond orientation is an accessible pedagogy and should still be taught, one could comment that there are reasons why this is not an absolute requirement.**

**Figure 21:**
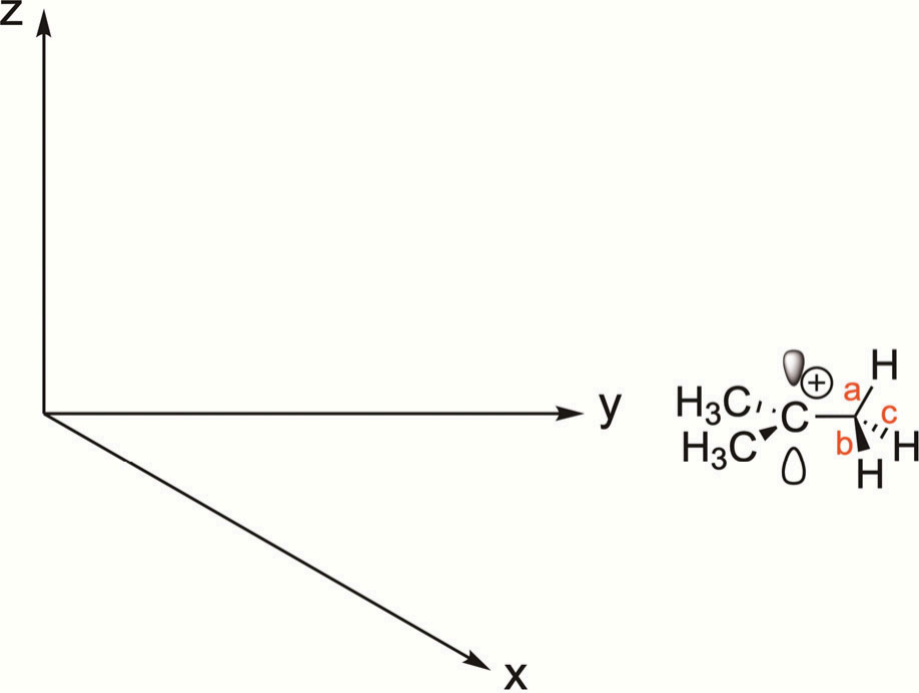
Alignment of *t*-butyl carbocation with Cartesian axes.

### A Simpler Pedagogy for Charge Stabilization?

The above pedagogies for charge stabilization (inductive, hyperconjugation, polarizability) consider how the charge, initially localized on one atom, is then stabilized by the attached group(s). This is a pedagogically sound approach, but not the only one we could take. We can also view this through the perspective of the canonical molecular orbitals that span the entire molecule such that the electrons are distributed throughout the molecule. A positive charge represents a net electron deficiency. Since all electrons are in orbitals spanning the molecule, the deficiency of an electron also spans the molecule. With a negative charge, the ‘excess’ electron is in an orbital spanning the molecule, so the same logic applies. We would not recommend teaching in this way to students, since a student in the early stages of their organic chemistry study would not know/predict the shapes of the canonical molecular orbitals of relevant charged structures. However, **it is useful for an educator to be aware of the broader context into which hyperconjugation fits**.

## Conclusion

We take the view that polarization is the primary ‘mechanism’ for charge stabilization by alkyl groups. Since we most commonly apply localized bonding models (i.e., hybridization), it is natural to seek a localized bonding representation for the charge stabilization. Hyperconjugation is most commonly represented using orbital diagrams that show it as a short-range specific orbital effect. However, the fact that we must introduce terms such as ‘double hyperconjugation’ (Figure 4) shows that consideration of extended electronic effects is required. Further examples of this are seen in organosilicon chemistry, since silicon stabilizes γ- and δ-carbocations by effects described as homohyperconjugation and double hyperconjugation.^76^ It may be simpler to state that silicon stabilizes carbocations since it is more polarizable than carbon; Lambert and co-workers connect hyperconjugation with polarizability in such systems.^76^ We have made a number of recommendations in this work, and these changes might simply be summarized as the need to be mindful that all molecules have delocalized molecular orbitals, and that these orbitals can be polarized by introduction of charge. Hyperconjugation (in all its variants) is what this polarization looks like when considered using localized bonding models. Therefore, we should not say that some of the stabilization of a carbocation (or alkoxide anion, etc.) is due to hyperconjugation and some is due to polarizability.

Not all polarizability effects should be conflated with hyperconjugation. For example, the acidity order in halogen acids (HI > HBr > HCl > HF) or that in EtSH > EtOH can be attributed to polarizability without the need for a hyperconjugation representation.

## Supplementary Material



## Data Availability

The computational data supporting the results presented in this article are freely available via the Cardiff University Data Catalogue (DOI: 10.17035/cardiff.29986519).

## Notes

This comprises graphs and discussion of other charge decomposition models (Hirshfeld, NPA, CHELPG, DDEC6) (PDF)
