# Supplementary material for: Exploring the Interface of Hyperconjugation and Polarizability—Enhancing Pedagogies for Charge Stabilization in Organic Chemistry
Source: J Chem Educ. 2026 Jul 28;103(8):4420–9. doi: 10.1021/acs.jchemed.6c00708 (PMC13480145; doi:10.1021/acs.jchemed.6c00708)
Supplement: Supplementary file 1 [file ed6c00708_si_001.pdf]

Exploring the Interface of Hyperconjugation and Polarizability – Enhancing Pedagogies for Charge Stabilization in Organic Chemistry

Mark C. Elliott*,[a] Kasimir P. Gregory,[b] Colan E. Hughes[a] and Benjamin D. Ward[a]

[a.] *School of Chemistry, Cardiff University, Park Place, Cardiff, CF10 3AT, UK.*
[b.] *School of Science and Technology, University of New England, Armidale, NSW 2351, Australia*

Benchmarking and General Discussion of Other Charge Models

The charge on an atom has no absolute definition, and there are therefore a multitude of ways of partitioning the total electron-density of a molecule between its individual atoms. One way to benchmark the calculated charges is to compare dipole moments from the density calculation with those calculated from the point charges. In the following Table, the best-performing charge decomposition method is highlighted green, with the second-best highlighted amber. Where the direction of the dipole moment from the point charges is significantly different to that from the density, this is shown in red.

Table S1: Dipole moments (in Debye) calculated from the electron density and from different charge partitioning methods (Hirshfeld, CM5, NPA, CHELPG, DDEC6), compared with available experimental values.

Compound	Dipole Moment (Density)	Hirshfeld	CM5	NPA	CHELPG	DDEC6	Experimental Dipole Moment
Hexane Field (0.005 a.u.)	1.1859	0.819	0.821	0.619	1.160	0.886	n/a
1-Fluoropentane (Field –0.02 a.u.)	2.3980*	1.557	1.503	2.127	2.294	1.410	n/a
1-Fluoropentane (Field –0.005 a.u.)	1.2142	0.862	0.994	3.288	1.258	1.545	n/a
1-Fluoropentane	2.1163	1.496	1.627	3.770	2.141	2.217	1.85[1]
1-Fluoropentane (Field +0.005 a.u.)	3.110	2.187	2.319	4.288	3.117	2.960	n/a
1-Fluoropentane (Field +0.02 a.u.)	6.3480	4.449	4.590	6.042	6.287	5.387	n/a
Pentylamine	1.4693	0.498	1.389	1.789	1.377	1.186	1.62[2]
Pentylammonium	11.4264	11.193	11.293	12.253	11.387	12.054	n/a
Methanol	1.7038	0.863	1.674	2.837	1.665	1.719	2.87[3]
Methoxide	2.4903	1.189	1.925	4.438	2.452	2.687	n/a
Ethanol	1.6157	0.787	1.558	2.721	1.559	1.700	1.69[4]
Ethoxide	4.1264	2.779	3.439	6.283	4.043	4.620	n/a
Pentan-1-ol	1.502	0.784	1.553	2.699	1.501	2.032	1.70[5]
Pentan-1-olate	12.2512	10.643	11.302		11.966	12.873	n/a

* The dipole direction is reversed compared to the other fluoropentane calculations.

[1] A. Bondi, Physical Properties of Molecular Crystals, Liquids, and Glasses, John Wiley, New York, 1968.
[2] B. Krishna and A. N. Srivastava, *J. Chem. Phys.*, 1962, **37**, 1574–1574.
[3] S. Terdale and A. Tantray, *J. Mol. Liquids*, 2017, **225**, 662–671.
[4] D. R. Lide (Ed.), *CRC Handbook of Chemistry and Physics*, 89th ed., CRC Press, Boca Raton, FL, 2008, pp. 9–55.
[5] A. G. Gilani, M. Moghadam and S. E. Hosseini, *J. Chem. Thermodyn.*, 2015, **91**, 384–395.

CHELPG charges consistently performed best in reproducing the dipole moment from the overall electron-density. Wiberg and Rablen noted that CHELPG charges “might best be considered as a set of empirical quantities that are designed to reproduce the electrostatic potential, rather than as charges in the usual sense.”[6] We note that CHELPG was co-developed by Wiberg,[7] who is presumably best placed to be aware of its limitations. In a more recent survey, Wiberg and Rablen give reasons for favouring Hirshfeld charges.[8] CM5 charges were designed to improve correlation with dipole moments,[9] so it is perhaps unsurprising that they perform better in this respect. We previously noted that it is possible for small changes in charge distribution to affect the dipole moment significantly,[10] such that the ability of a charge model to reproduce a dipole moment from point charges is not necessarily a measure of success of that charge model. On this basis, while CM5 charges are highly regarded, it is more important that identical conclusions are reached with a selection of charge models (in the case of this study, Hirshfeld, CM5, NPA, CHELPG and DDEC6) than to try to objectively determine which charge model best represents the distribution of electron-density between atoms. We do, however, echo Wiberg’s comments in the validity of CHELPG charges. The chart below shows the amalgamated charge on methyl or methylene groups in hexane using the five different charge models.

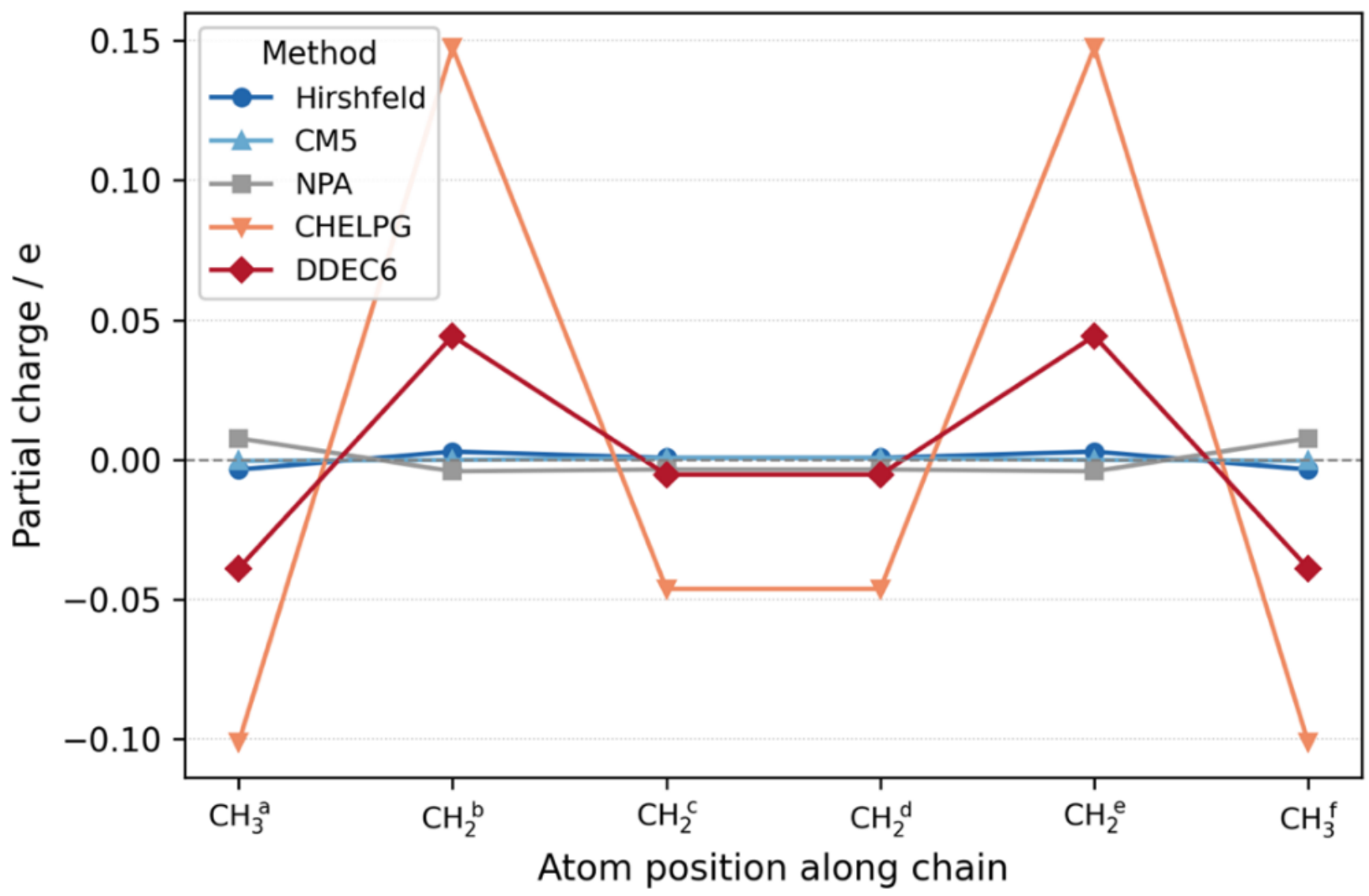

Figure S1: Group charges in *n*-hexane calculated with different charge partitioning methods. The plotted values correspond to the net partial charges on each methyl (CH_3) or methylene (CH_2) group, as indicated in the molecular structure inset.

The CHELPG charges are considerably out of step with the other charges. The CH_3 groups have a charge that is 0.25 e more negative than the adjacent CH_2 groups, which appears highly counter-intuitive. There is no obvious reason for this bond polarization. DDEC6 charges exhibit a similar trend, though to a much smaller extent, which is consistent with their design as a density-based method that balances electrostatic accuracy with chemical reasonableness.

[6] K. B. Wiberg and P. R. Rablen, *J. Comp.*, 1993, **14**, 1504–1518.
[7] C. M. Breneman and K. B. Wiberg, *J. Comp. Chem.*, 1990, **11**, 361–373.
[8] K. B. Wiberg and P. R. Rablen, *J. Org. Chem.*, 2018, **83**, 15463–15469.
[9] A. V. Marenich, S. V. Jerome, C. J. Cramer and D. G. Truhlar, *J. Chem. TheoryComput.*, 2012, **8**, 527–541.
[10] M. C. Elliott, C. E. Hughes, P. J. Knowles and B. D. Ward, *Org. Biomol. Chem.*, 2025, **23**, 352–359.

Hexane – Consideration of Other Charge Models

The data from **Figure 7** (change in charge as a result of applied field) in the manuscript are shown graphically below, including four other charge models. Despite our concerns about CHELPG charges (above) we find the same general trend – there is a larger change in charge for the methyl groups compared to the methylene groups. The other charge models considered are in remarkable agreement with respect to the amount of change in calculated charge as a result of the computationally applied field. That is, our conclusions do not depend on the choice of charge decomposition method.

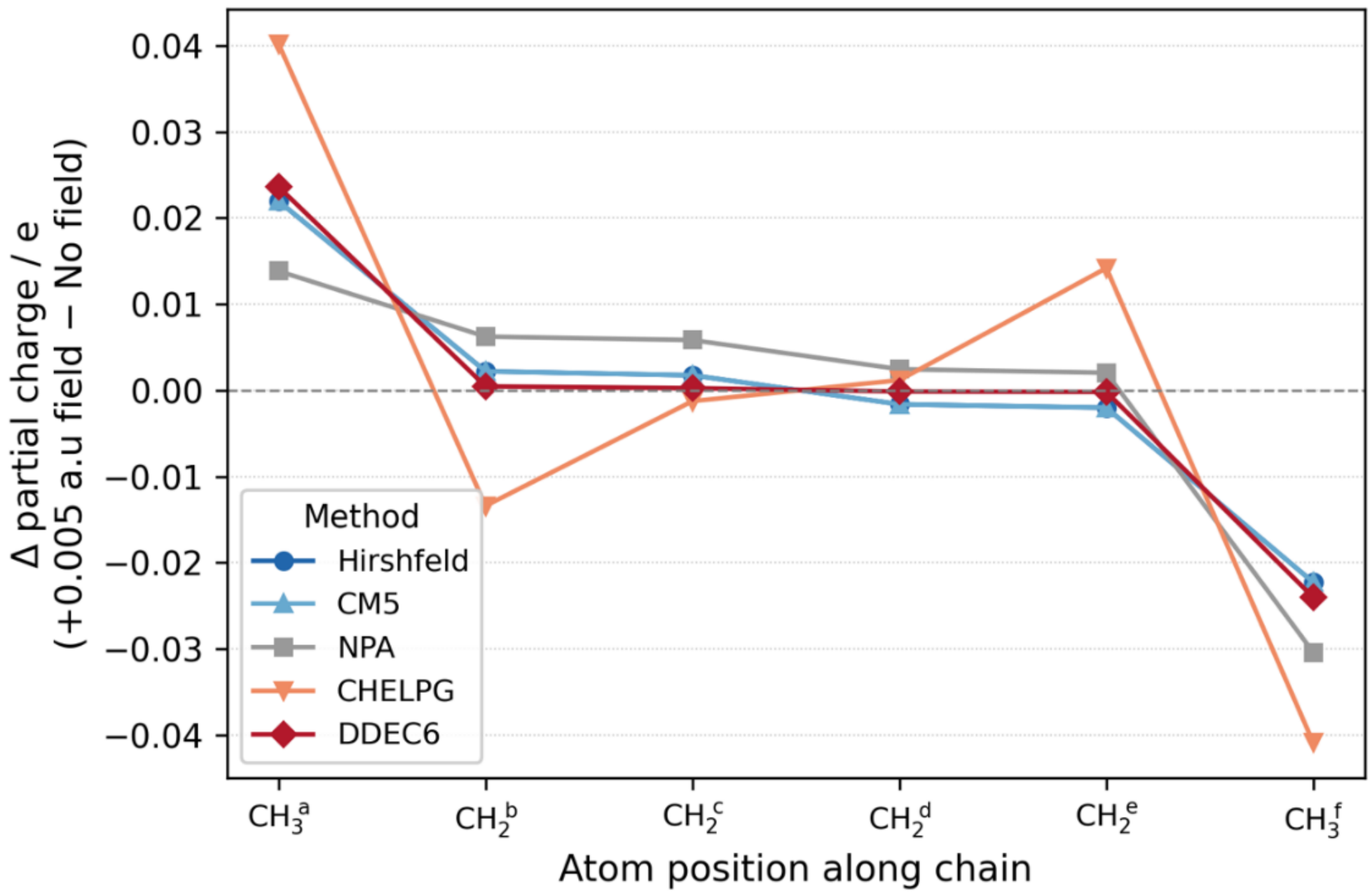

Figure S2: Change in group charges of *n*-hexane upon application of an external field (+0.005 a.u.) relative to the no-field case, calculated using different charge partitioning methods. Plotted values correspond to the difference in partial charge for each methyl (CH_3) and methylene (CH_2) group along the chain, as labelled in the molecular structure inset.

NBO Interactions (kcal mol^{-1}) in Hexane in the Absence and Presence of an Applied Field (0.005 a.u.)

Table S2. Natural bond orbital (NBO) second-order perturbation stabilization energies (in kcal mol^{-1}) for hyperconjugative donor–acceptor interactions in hexane, calculated in the absence and presence of an applied electric field (+0.005 a.u.). Positive differences correspond to enhanced stabilization under the field, while negative values indicate reduced stabilization. The applied field drives electron density in the direction from H3 toward H19, increasing interactions in this direction and decreasing those in the reverse direction.

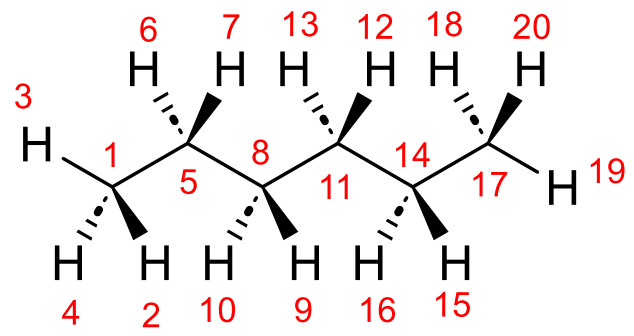

Donor (σ)	Acceptor (σ*)	No field	Field (+0.005 a.u.)	Difference
C1–H3	C5–C8	3.60	3.81	+0.21
C1–C5	C8–C11	2.59	2.76	+0.17
C5–C8	C11–C14	2.41	2.57	+0.16
C8–C11	C14–C17	2.57	2.77	+0.20
C11–C14	C17–H19	1.99	2.16	+0.17
C17–H19	C11–C14	3.60	3.39	–0.21
C14–C17	C8–C11	2.59	2.43	–0.16
C11–C14	C5–C8	2.41	2.26	–0.15
C8–C11	C1–C5	2.57	2.39	–0.18
C5–C8	C1–H3	1.99	1.83	–0.16
C1–H2	C5–H6	3.72	3.83	+0.11
C1–H4	C5–H7	3.72	3.83	+0.11
C5–H6	C8–H9	4.20	4.31	+0.11
C5–H7	C8–H10	4.20	4.31	+0.11
C8–H9	C11–H13	4.14	4.29	+0.15
C8–H10	C11–H12	4.14	4.29	+0.15
C11–H12	C14–H16	3.92	4.04	+0.12
C11–H13	C14–H15	3.92	4.04	+0.12
C14–H15	C17–H18	3.77	3.93	+0.16
C14–H16	C17–H20	3.77	3.93	+0.16
C17–H18	C14–H15	3.72	3.61	–0.11
C17–H20	C14–H16	3.72	3.61	–0.11
C14–H15	C11–H13	4.20	4.10	–0.10
C14–H16	C11–H12	4.20	4.10	–0.10
C11–H12	C8–H10	4.14	4.01	–0.13
C11–H13	C8–H9	4.14	4.01	–0.13
C8–H9	C5–H6	3.92	3.81	–0.11
C8–H10	C5–H7	3.92	3.81	–0.11
C5–H6	C1–H2	3.77	3.62	–0.15
C5–H7	C1–H4	3.77	3.62	–0.15

Fluoropentane

The trends in Hirshfeld and NPA charges follow those of CM5 charges as described in the main manuscript.

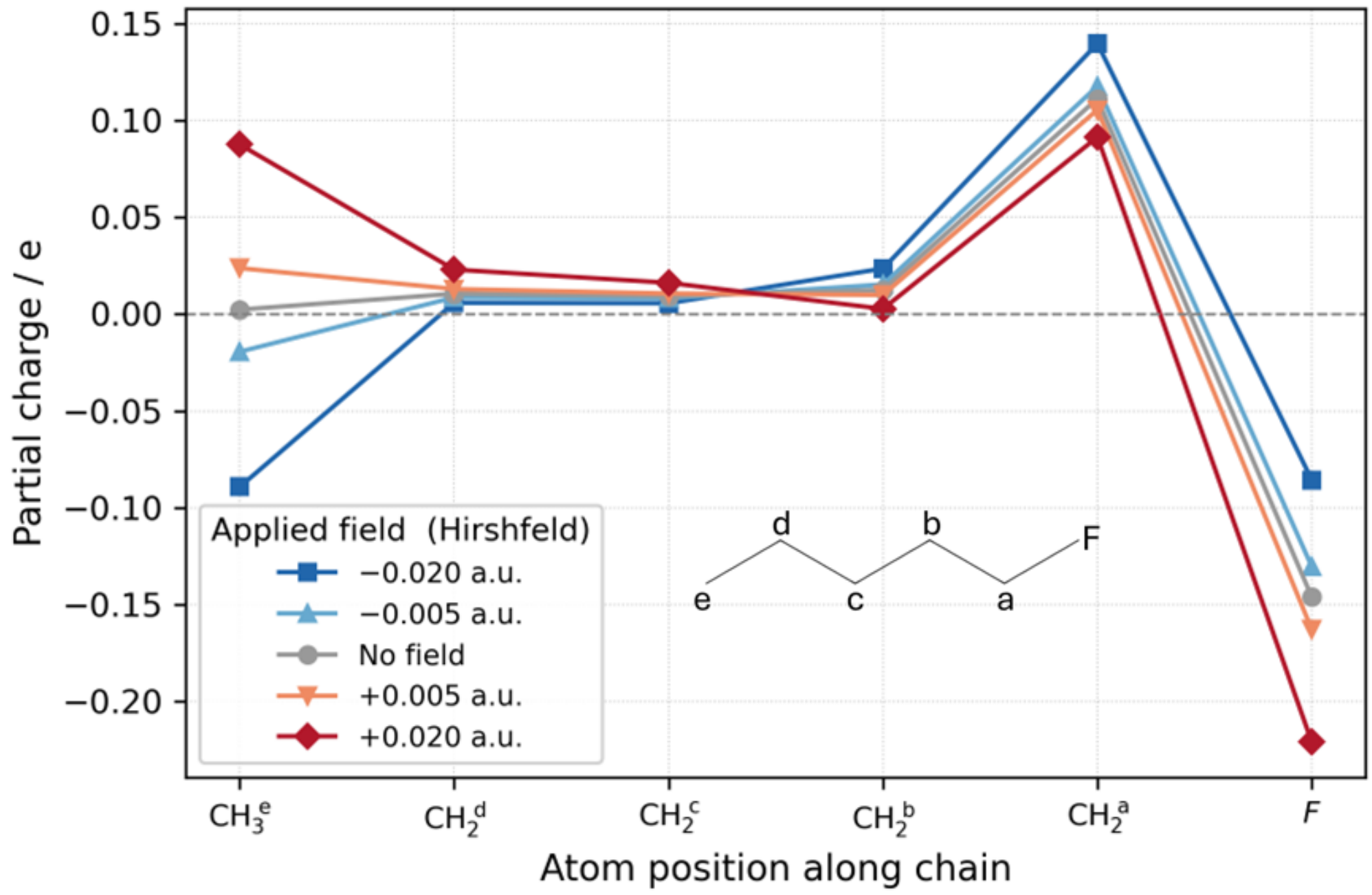

Figure S3: Hirshfeld group charges in 1-fluoropentane under different applied electric fields (–0.020, –0.005, 0, +0.005, and +0.020 a.u.). Values correspond to the net partial charges of each methyl (CH_3), methylene (CH_2), and fluorine atom along the chain, as indicated in the molecular structure inset.

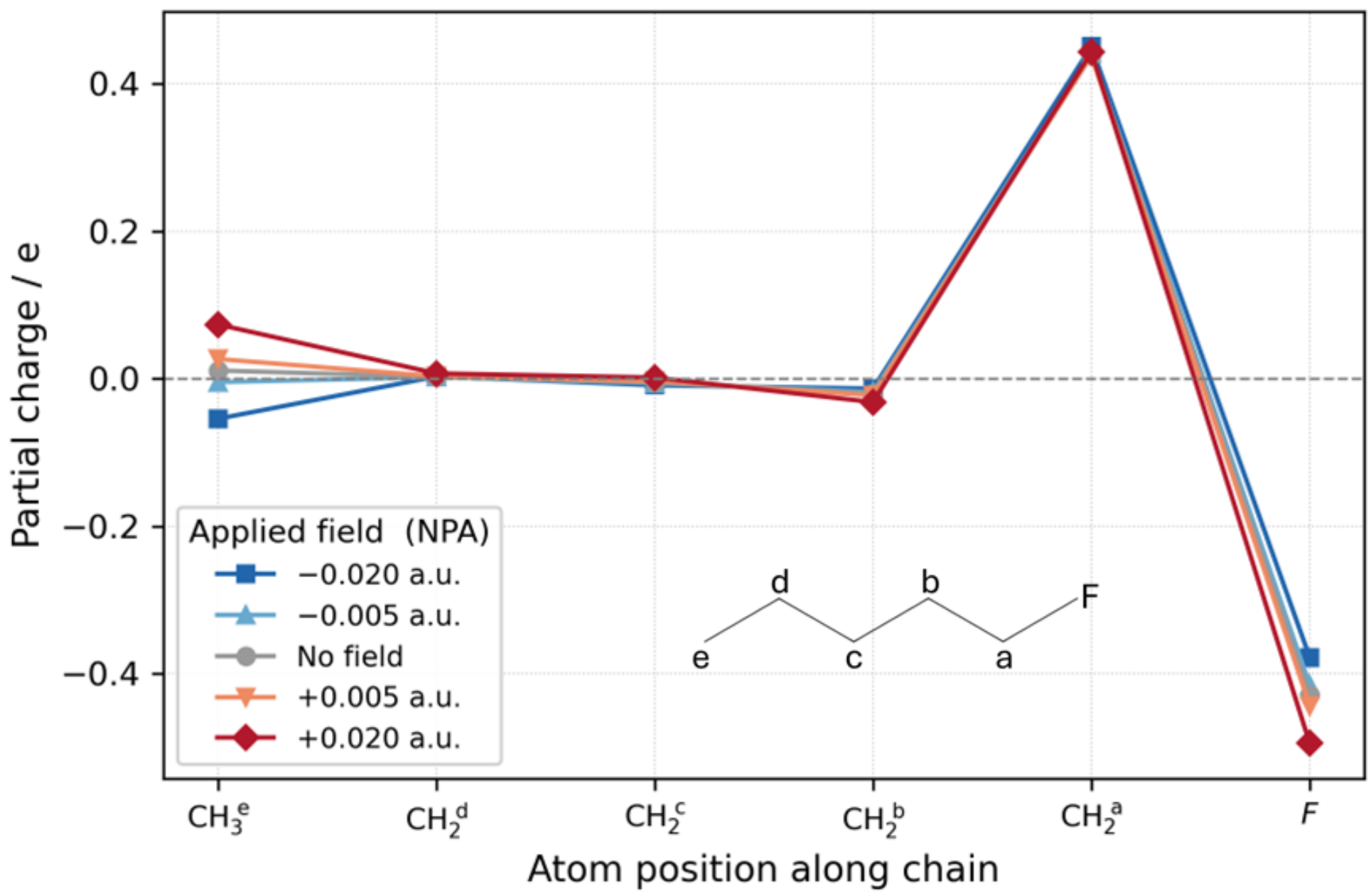

Figure S4: NPA group charges in 1-fluoropentane under different applied electric fields (–0.020, –0.005, 0, +0.005, and +0.020 a.u.). Values correspond to the net partial charges of each methyl (CH_3), methylene (CH_2), and fluorine atom along the chain, as indicated in the molecular structure inset.

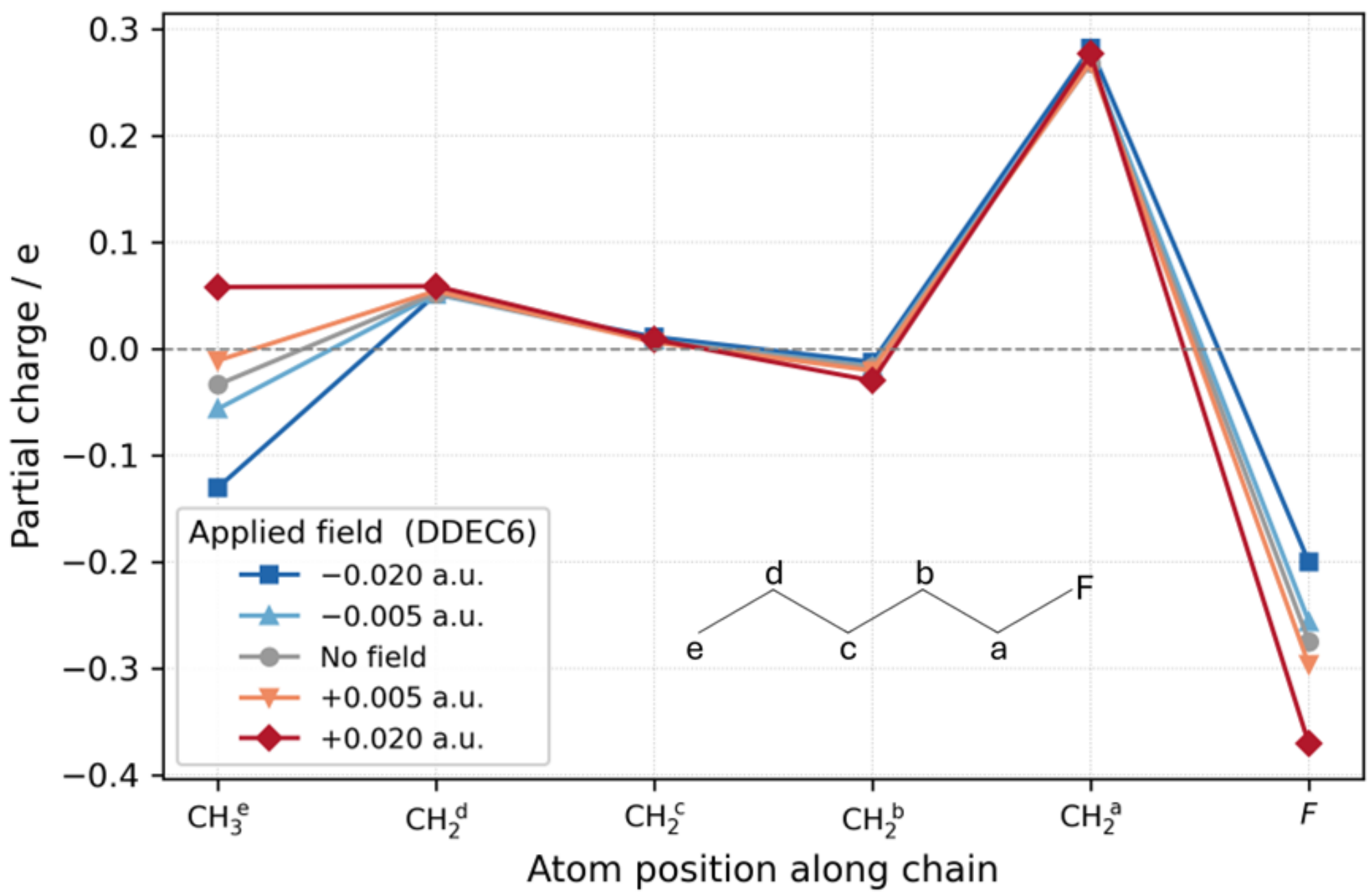

Figure S5: DDEC6 group charges in 1-fluoropentane under different applied electric fields (–0.020, –0.005, 0, +0.005, and +0.020 a.u.). Values correspond to the net partial charges of each methyl (CH_3), methylene (CH_2), and fluorine atom along the chain, as indicated in the molecular structure inset. With DDEC6 charges, fluorine is found to be more electron-withdrawing from C4 than from C2 or C3, which is surprising. However, the applied field induces the same effect as seen with the other charge models.

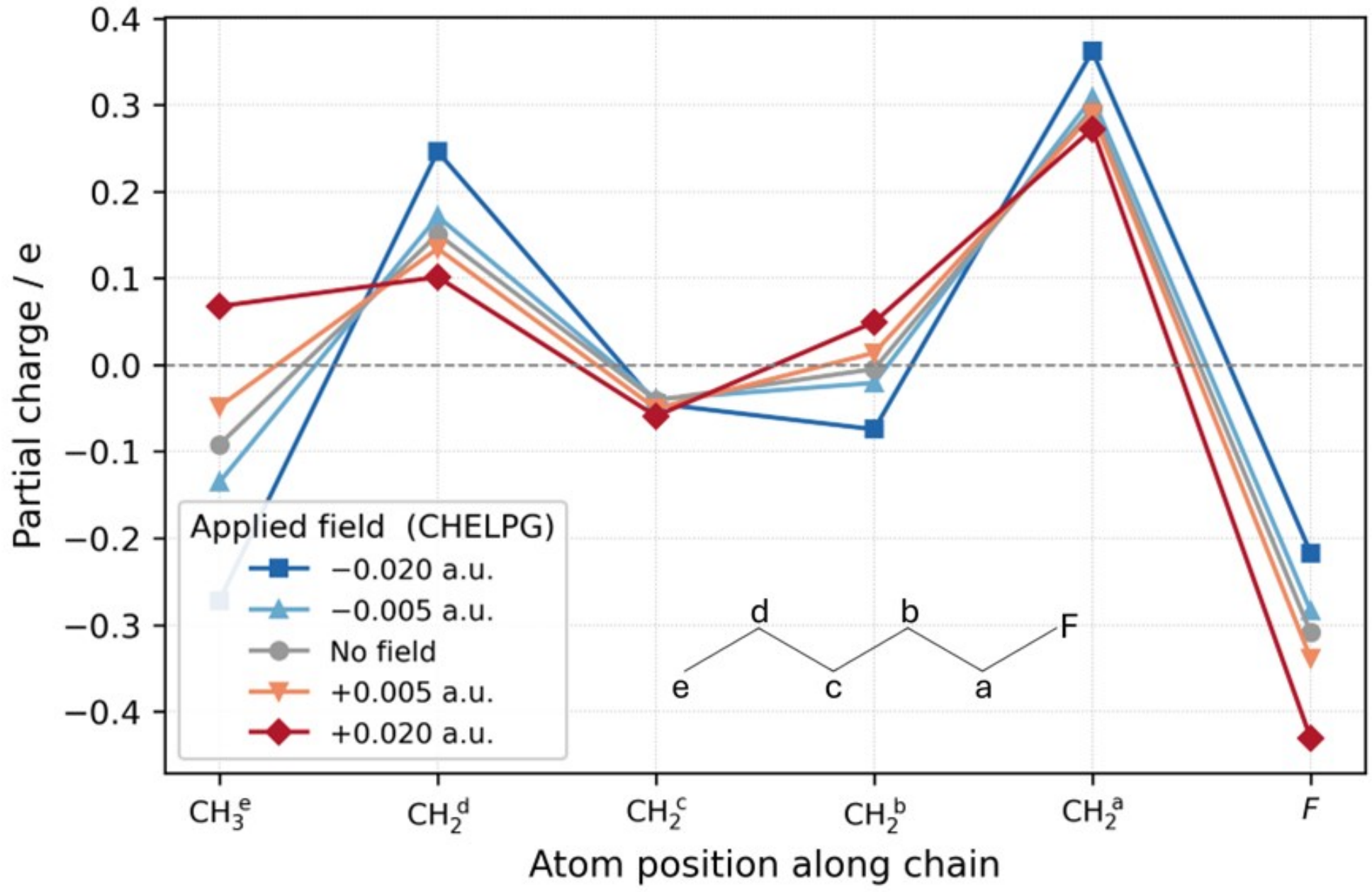

Figure S6: CHELPG group charges in 1-fluoropentane under different applied electric fields (–0.020, –0.005, 0, +0.005, and +0.020 a.u.). Values correspond to the net partial charges of each methyl (CH_3), methylene (CH_2), and fluorine atom along the chain, as indicated in the molecular structure inset. As with hexane, there is a larger variation in CHELPG charges for the methylene groups. Broadly, we find that the applied field induces a transfer of electron-density between the CH_3 group and fluorine, but the changes at the intervening atoms are less straightforward.

NBO Interactions (kcal mol^{-1}) in Fluoropentane

Table S3: Natural bond orbital (NBO) second-order perturbation stabilization energies (in kcal mol^{-1}) for donor–acceptor interactions in 1-fluoropentane under different applied electric fields (+0.020, +0.005, 0, –0.005, –0.020 a.u.).

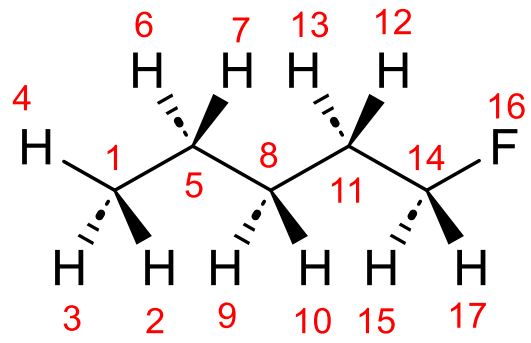

Donor (σ or LP)	Acceptor (σ*)	Field/a.u. +0.02	+0.005	No field	–0.005	–0.02
C1–H2	C5–H6	4.10	3.82	3.72	3.61	3.25
C1–H3	C5–H7	4.10	3.82	3.72	3.61	3.25
C1–H4	C5–C8	4.54	3.88	3.67	3.49	2.97
C1–C5	C8–C11	3.54	2.82	2.60	2.41	1.88
C5–H6	C1–H2	3.17	3.59	3.73	3.89	4.40
C5–H6	C8–H10	4.56	4.28	4.18	4.08	3.79
C5–H7	C1–H3	3.17	3.59	3.73	3.89	4.40
C5–H7	C8–H9	4.56	4.28	4.18	4.08	3.79
C5–C8	C1–H4	1.43	1.80	1.95	2.10	2.65
C5–C8	C11–C14	2.52	2.31	2.23	2.14	1.94
C8–H9	C5–H7	3.34	3.70	3.80	3.91	4.19
C8–H9	C11–H12	4.22	3.99	3.88	3.75	3.37
C8–H10	C5–H6	3.34	3.70	3.80	3.91	4.19
C8–H10	C11–H13	4.22	3.99	3.88	3.75	3.37
C8–C11	C1–C5	1.89	2.39	2.59	2.81	3.58
C8–C11	C14–F16	5.99	3.77	3.25	2.82	1.74
C11–H12	C8–H9	3.42	3.87	4.01	4.15	4.59
C11–H12	C14–H15	3.94	3.56	3.43	3.31	2.92
C11–H13	C8–H10	3.42	3.87	4.01	4.15	4.59
C11–H13	C14–H17	3.94	3.56	3.43	3.31	2.92
C11–C14	C5–C8	2.04	2.38	2.49	2.61	2.96
C14–H15	C11–H12	3.51	3.53	3.54	3.56	3.59
C14–F16	C8–C11	0.94	1.20	1.30	1.42	1.79
C14–H17	C11–H13	3.51	3.53	3.54	3.56	3.59
F16 lone pair 1	C8–C11	0.63	0.90	1.00	1.11	7.40
F16 lone pair 1	C11–C14	5.19	6.28	6.62	6.91	7.40
F16 lone pair 1	C14–H15	1.67	2.03	2.13	2.20	1.50
F16 lone pair 1	C14–H17	1.67	2.03	2.13	2.20	7.97
F16 lone pair 2	C14–H15	4.73	5.92	6.26	6.55	2.42
F16 lone pair 2	C14–H17	4.73	5.92	6.26	6.55	2.42

Stabilization of Alkoxide Anions

Table S4: Sum of electronic and thermal free energies (Hartree) of alcohols and their corresponding alkoxides, with differences reported in kcal mol^{-1}. Relative differences are given with respect to methanol/methoxide. The small variations across substituents (Me, Et, *n*-C_5H_{11}) indicate that deprotonation energies are relatively insensitive to alkyl chain length. In line with the known gas phase acidity of alcohols,[11] we find that the calculated free energy for the formation of alkoxides from alcohols decreases in the order Me > Et > *n*-C_5H_{11}.

R =	Alcohol	Alkoxide	Difference / kcal mol^{-1}	Relative difference / kcal mol^{-1}
Me	–115.4999	–114.89639	378.7	0
Et	–154.70302	–154.10467	375.5	3.2
n-C_5H_{11}	–272.29827	–271.70231	374.0	4.7

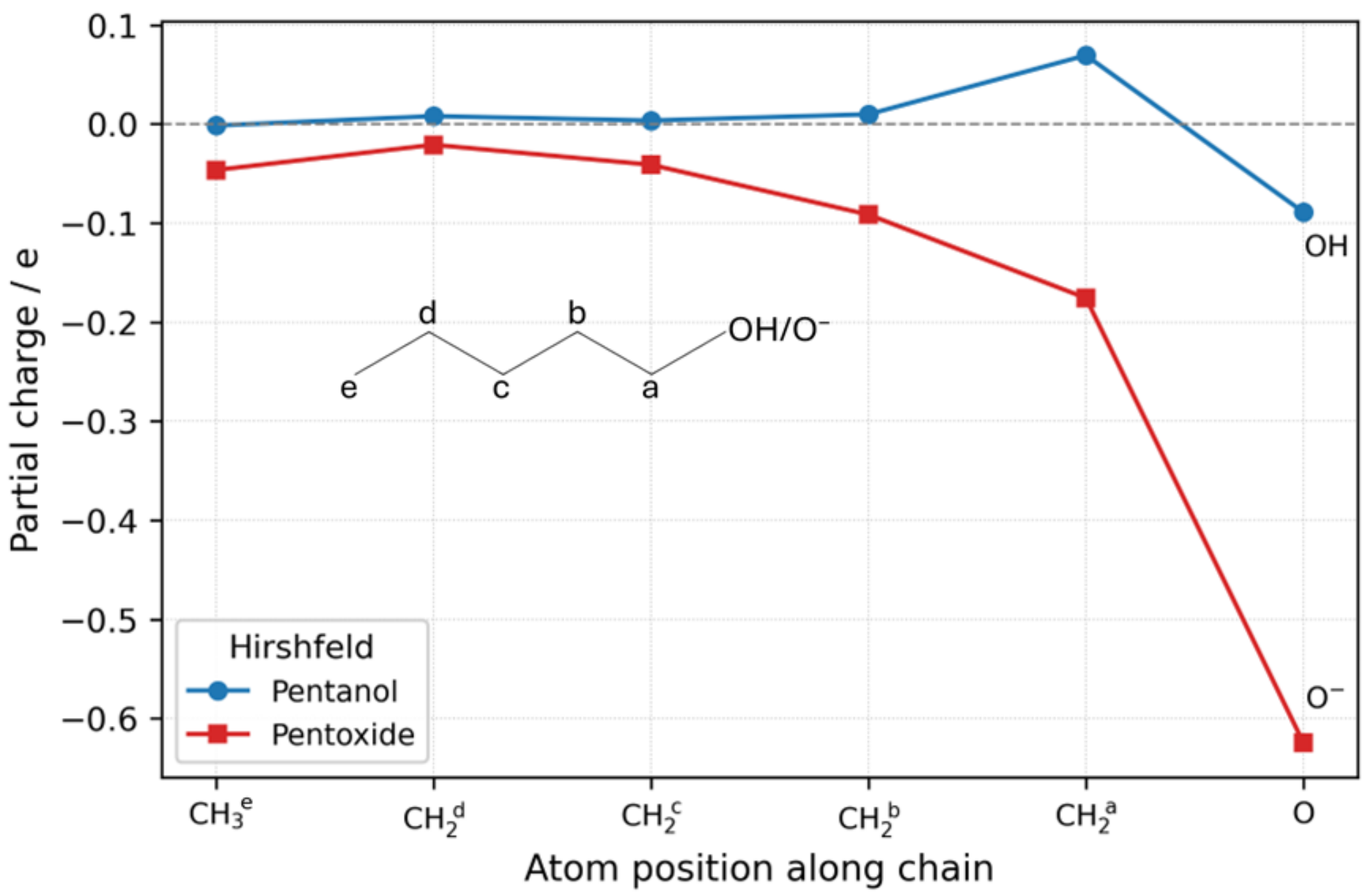

Figure S7. Hirshfeld group charges in pentanol and pentoxide. Values correspond to the net partial charges of each CH_3, CH_2, and terminal OH/O^- group along the chain, as indicated in the molecular structure inset. Deprotonation to form the alkoxide results in a substantial buildup of negative charge localized on the oxygen atom, with smaller delocalization of charge along the alkyl chain. Hirshfeld charges show the same as the CM5 charges in the main manuscript.

[11] G. Boand, R. Houriet and T. Gäumann, *J. Am. Chem. Soc.*, 1983, **105**, 2203–2206.

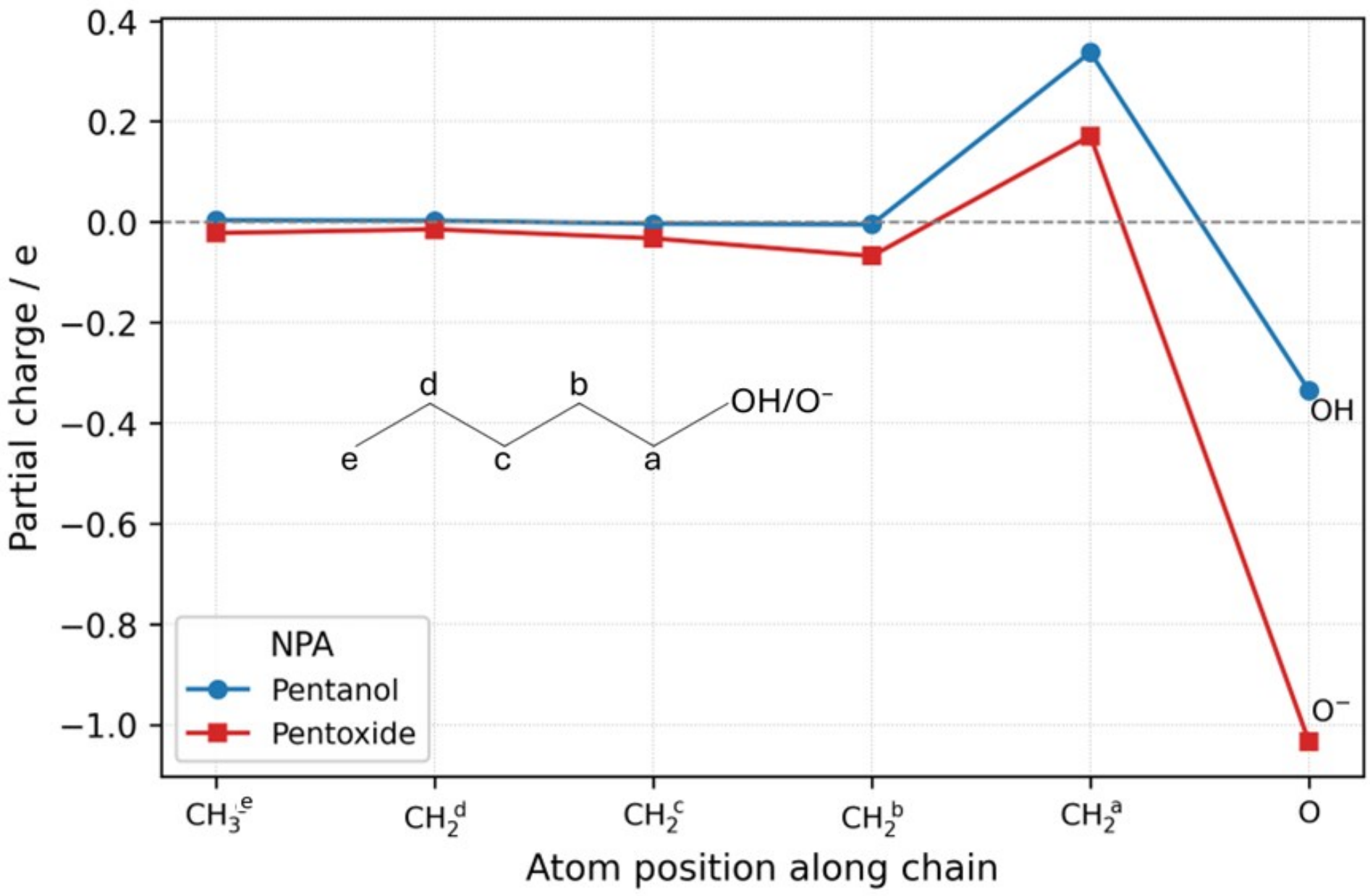

Figure S8. NPA group charges in pentanol and pentoxide. Values correspond to the net partial charges of each CH_3, CH_2, and terminal OH/O^- group along the chain, as indicated in the molecular structure inset. Deprotonation to form the alkoxide results in a substantial buildup of negative charge localized on the oxygen atom, with smaller delocalization of charge along the alkyl chain.

The differences in DDEC6 charges between pentanol and pentoxide are again in line with the CM5 charges, although in this case, both have more electron-withdrawal from C4 than from C3.

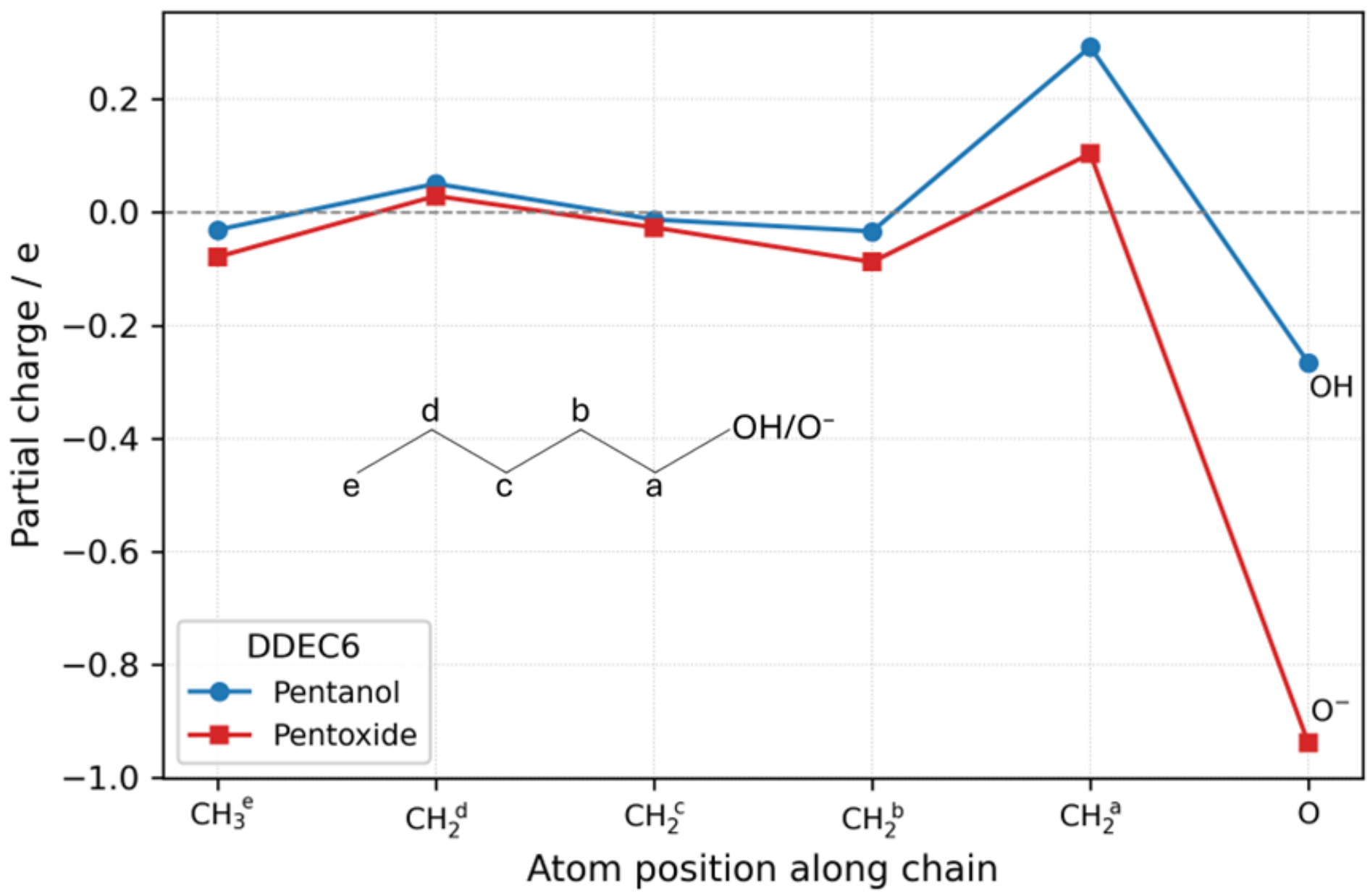

Figure S9. DDEC6 group charges in pentanol and pentoxide. Values correspond to the net partial charges of each CH_3, CH_2, and terminal OH/O^- group along the chain, as indicated in the molecular structure inset. Deprotonation to form the alkoxide results in a substantial buildup of negative charge localized on the oxygen atom, with smaller delocalization of charge along the alkyl chain.

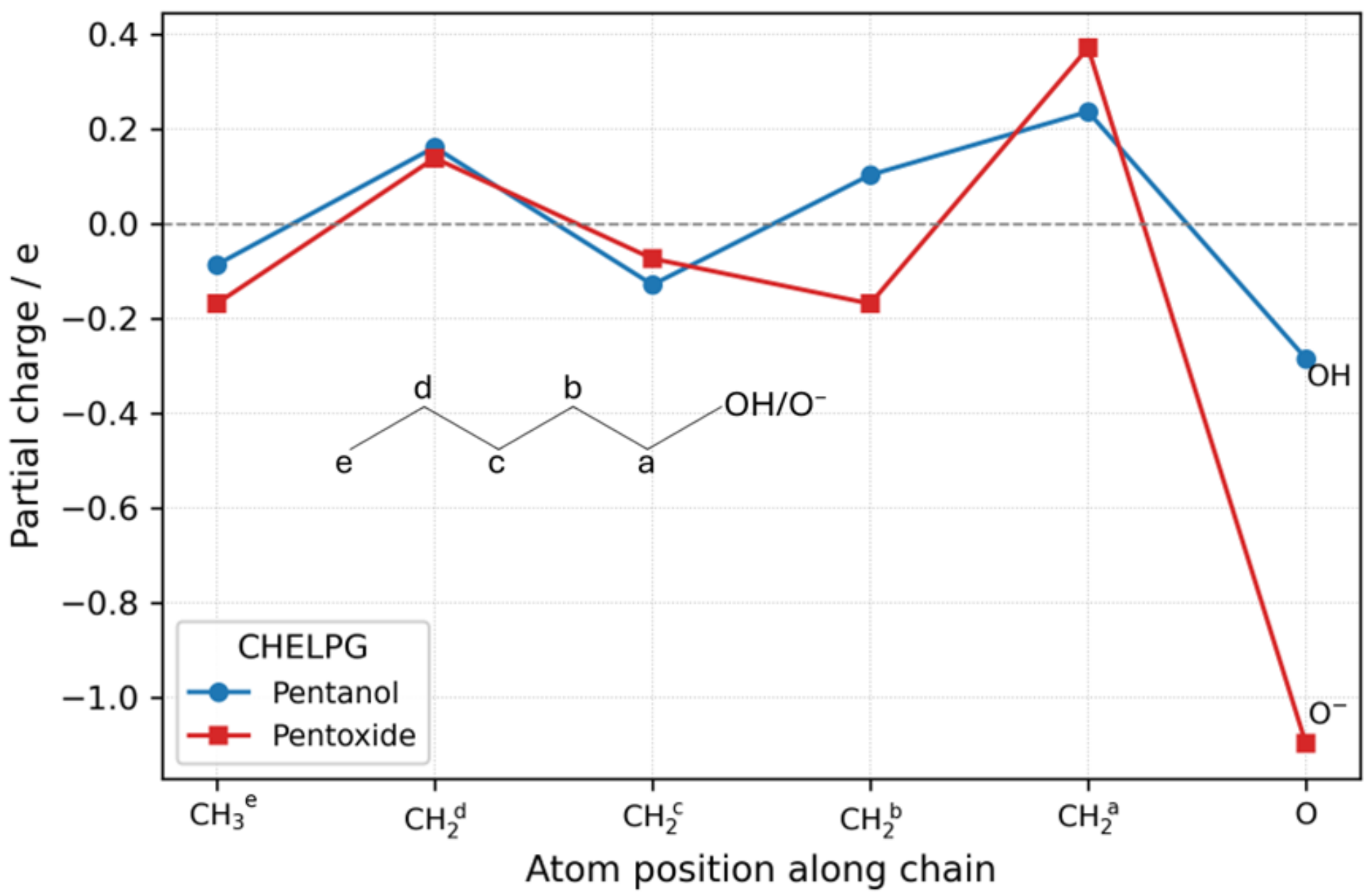

Figure S10. CHELPG group charges in pentanol and pentoxide. Values correspond to the net partial charges of each CH_3, CH_2, and terminal OH/O^- group along the chain, as indicated in the molecular structure inset. Deprotonation to form the alkoxide results in a substantial buildup of negative charge localized on the oxygen atom, with smaller delocalization of charge along the alkyl chain. CHELPG once again is the least consistent with the other charge decomposition analysis methods. With the CHELPG charges, the immediate effect at CH_2^a is confusing. Given Wiberg's observations, we will make no further comment.

Selected NBO Interactions (kcal mol^{-1}) in Pentan-1-ol and 1-Pentoxide

Table S5: NBO interactions involving the oxygen lone pairs, which are all larger in the pentoxide anion.

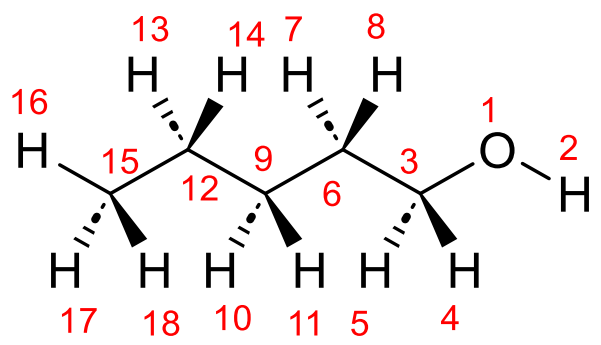

Donor	Acceptor (σ*)	Pentanol	Pentoxide	Difference
O LP 1	C3–H4	8.27	20.64	+12.37
O LP 1	C3–H5	8.27	20.64	+12.37
O LP 2	C3–C6	1.13	20.6	+19.47
O LP 2	C3–C6	1.13	20.6	+19.47
O LP 2	C3–H4	0.94	7.45	+6.51
O LP 2	C3–H5	0.94	7.45	+6.51
O LP 2	C6–C9	0.86	1.97	+1.11

Table S6: NBO second-order perturbation stabilization energies (kcal mol^{-1}) for *anti* vicinal σ→σ interactions along the pentanol chain and the corresponding pentoxide anion. Apart from the O1–C3 → C6–C9 interaction, all interactions are strengthened in the anion, consistent with enhanced electron donation from the negatively charged oxygen.

Donor (σ)	Acceptor (σ*)	Pentanol	Pentoxide	Difference
O1–C3	C6–C9	1.92	1.55	–0.37
C3–C6	C9–C12	2.67	3.09	+0.42
C6–C9	C12–C15	2.88	3.06	+0.18
C9–C12	C15–H16	2.23	2.26	+0.03
C3–H4	C6–H7	3.76	4.82	+1.06
C3–H5	C6–H8	3.76	4.82	+1.06
C6–H7	C9–H11	4.31	4.79	+0.48
C6–H8	C9–H10	4.31	4.79	+0.48
C9–H10	C12–H14	4.03	4.36	+0.33
C9–H11	C12–H13	4.03	4.36	+0.33
C12–H13	C15–H18	4.00	4.09	+0.09
C12–H14	C15–H17	4.00	4.09	+0.09

Table S7: NBO second-order perturbation stabilization energies (kcal mol^{-1}) for σ→σ interactions directed toward the oxygen substituent in pentanol and pentoxide. All interactions are weakened upon deprotonation, consistent with the reduced ability of the pentoxide anion to accept hyperconjugative donation along the chain.

**Donor (σ)**	**Acceptor (σ*)**	**Pentanol (kcal mol^{-1})**	**Pentoxide (kcal mol^{-1})**	**Difference (kcal mol^{-1})**
C15–H16	C9–C12	3.96	3.28	–0.68
C12–C15	C6–C9	2.79	2.26	–0.53
C9–C12	C3–C6	2.43	1.83	–0.60
C6–C9	C1–C3	2.93	1.81	–1.12
C15–H17	C12–H14	3.79	3.61	–0.18
C15–H18	C12–H13	3.79	3.61	–0.18
C12–H13	C9–H11	4.38	4.06	–0.32
C12–H14	C9–H10	4.38	4.06	–0.32
C9–H10	C6–H8	4.08	3.99	–0.09
C9–H11	C6–H7	4.08	3.99	–0.09
C6–H7	C3–H4	3.64	3.11	–0.53
C6–H8	C3–H5	3.64	3.11	–0.53

Pentylammonium

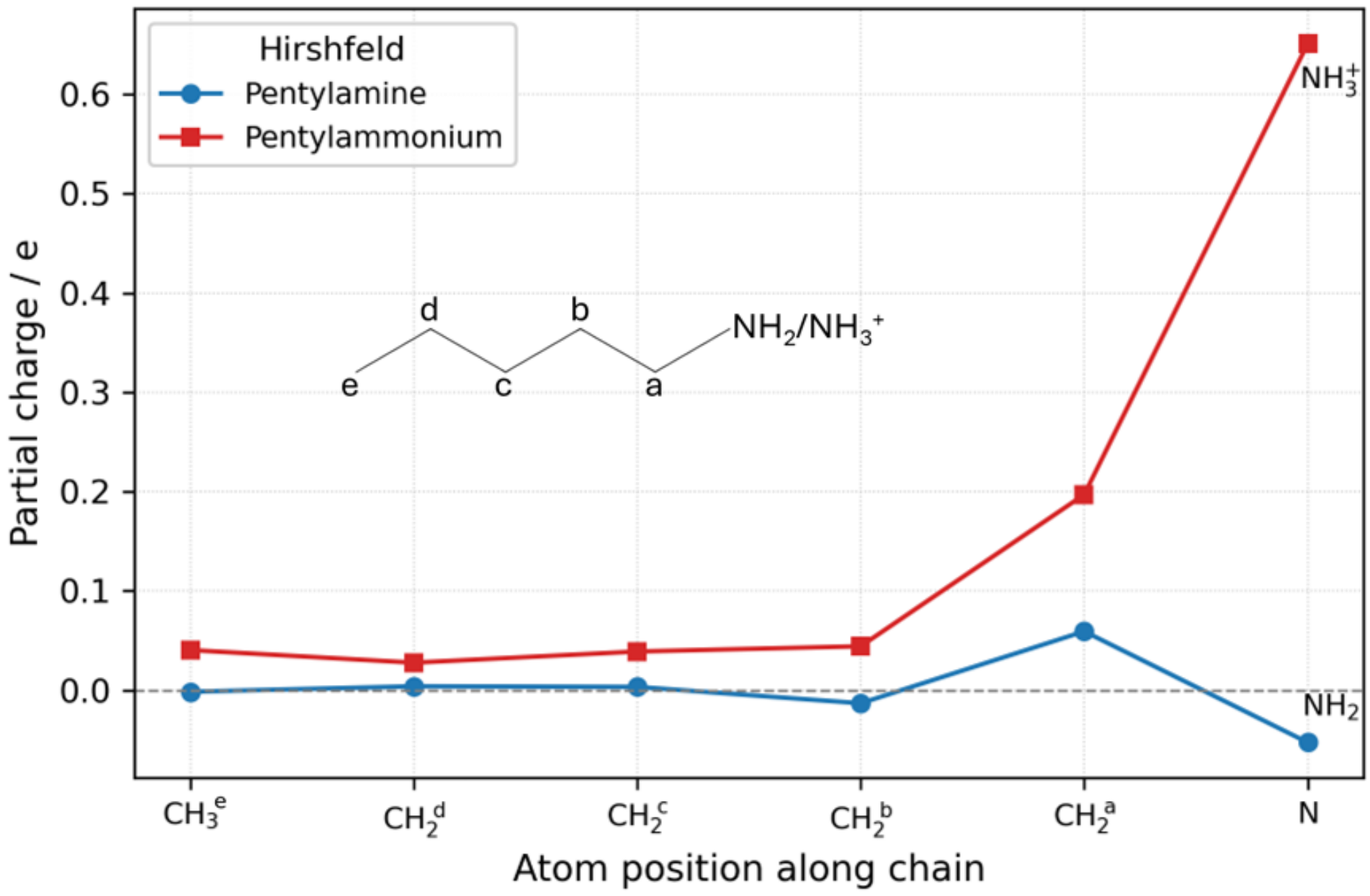

Figure S11: Hirshfeld group charges in pentylamine and pentylammonium. Values correspond to the net partial charges of each CH_3, CH_2, and terminal NH_2/NH_3^+ group along the chain, as indicated in the molecular structure inset. Protonation to form pentylammonium leads to a pronounced increase in positive charge localized on the ammonium group, with smaller but systematic increases across the alkyl chain. This is consistent with the CM5 distribution.

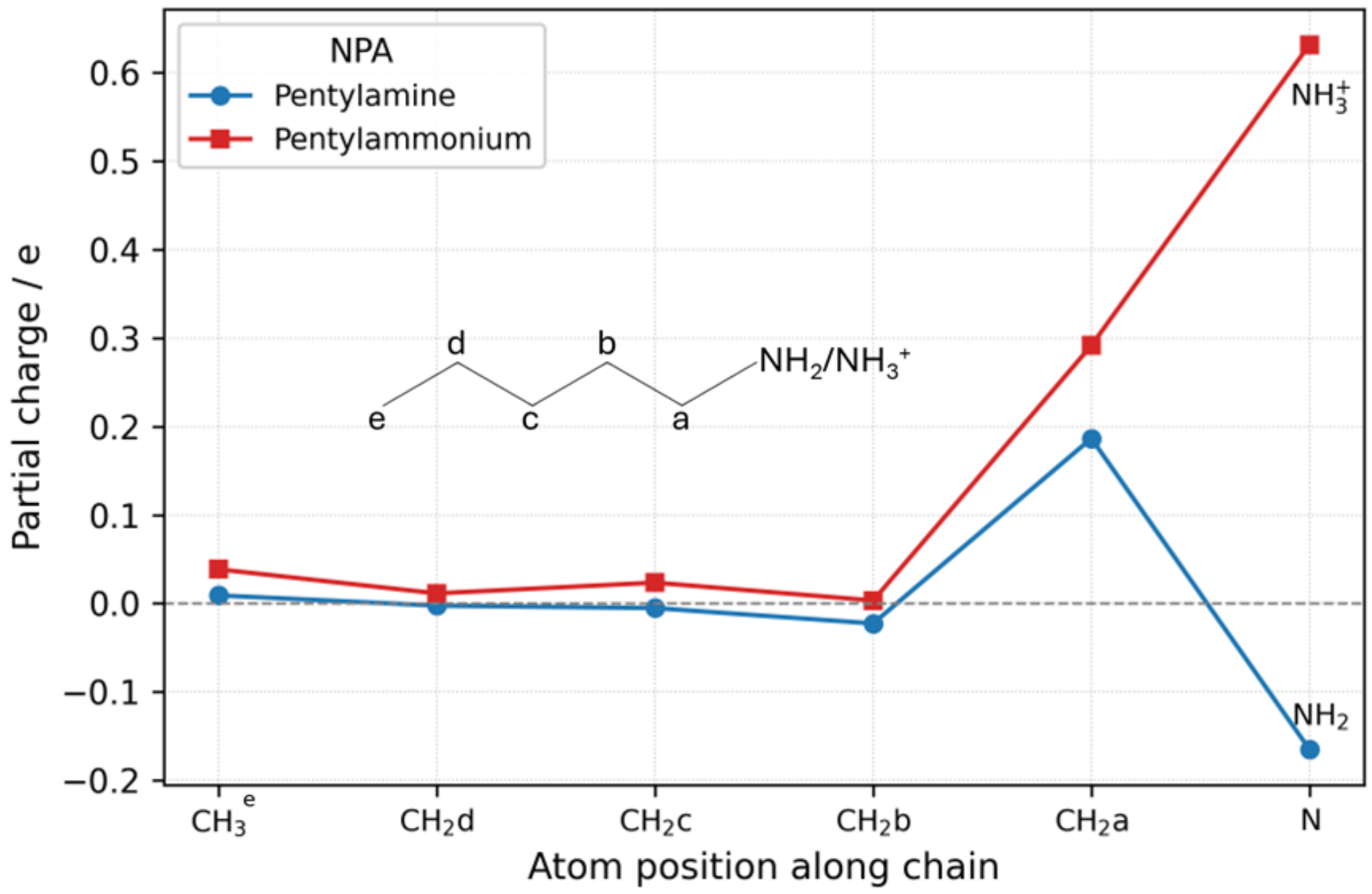

Figure S12: NPA group charges in pentylamine and pentylammonium. Values correspond to the net partial charges of each CH_3, CH_2, and terminal NH_2/NH_3^+ group along the chain, as indicated in the molecular structure inset. Protonation to form pentylammonium leads to a pronounced increase in positive charge localized on the ammonium group, with smaller but systematic increases across the alkyl chain. This is consistent with the CM5 distribution.

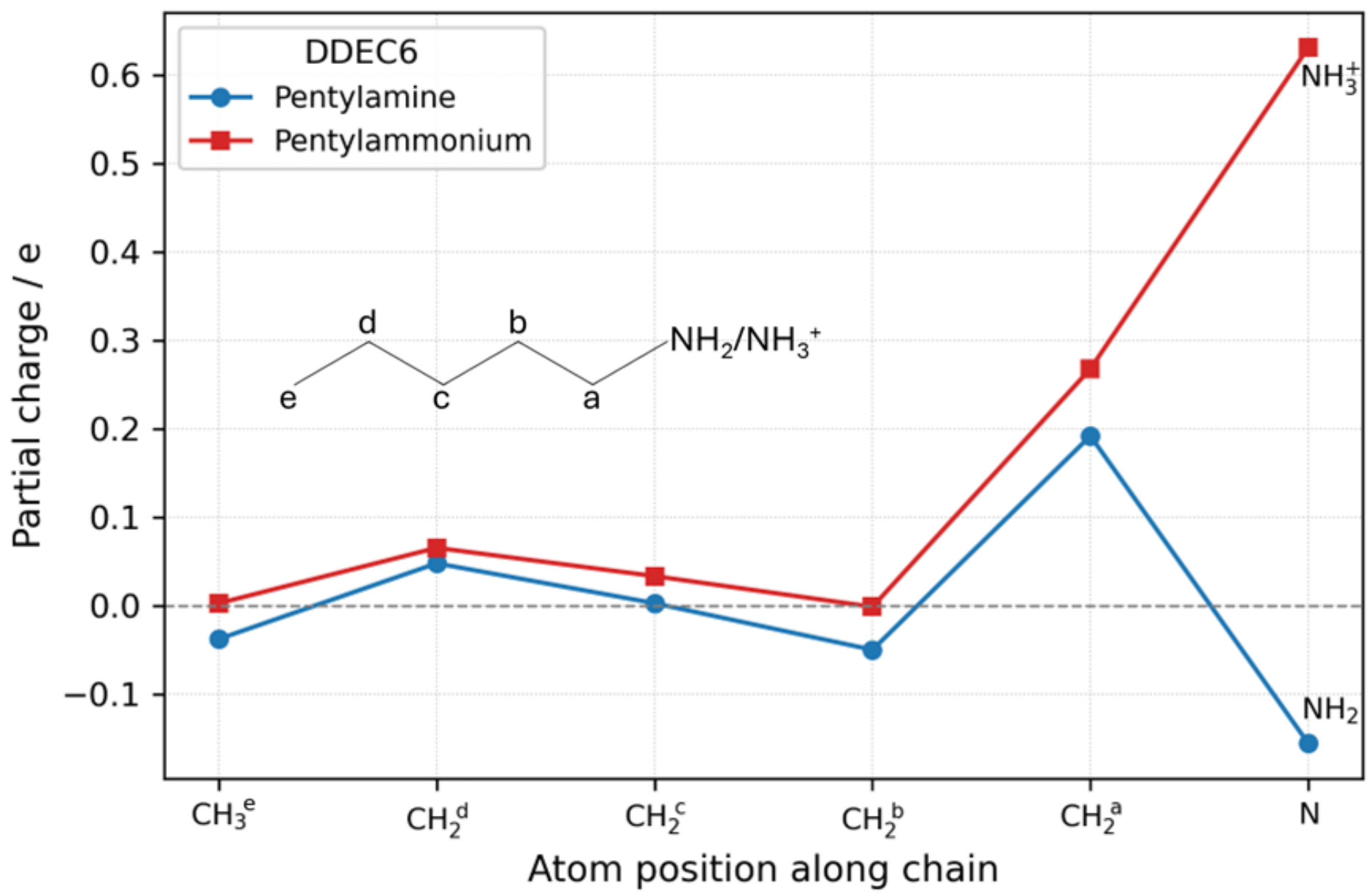

Figure S13: DDEC6 group charges in pentylamine and pentylammonium. Values correspond to the net partial charges of each CH_3, CH_2, and terminal NH_2/NH_3^+ group along the chain, as indicated in the molecular structure inset. Protonation to form pentylammonium leads to a pronounced increase in positive charge localized on the ammonium group. This is mostly consistent with the CM5 distribution; however there is more diversity of charge through the alkyl chain.

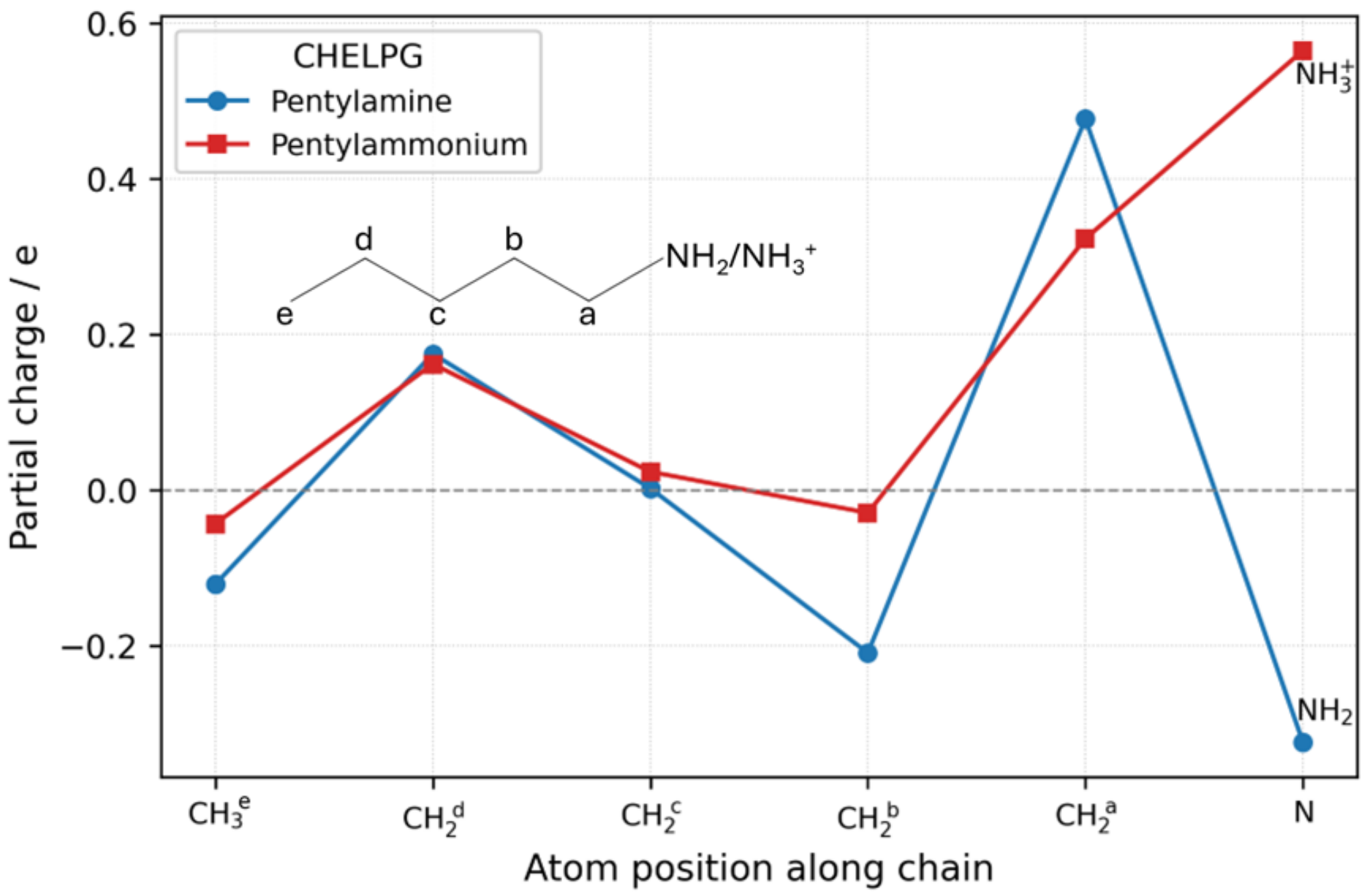

Figure S14: CHELPG group charges in pentylamine and pentylammonium. Values correspond to the net partial charges of each CH_3, CH_2, and terminal NH_2/NH_3^+ group along the chain, as indicated in the molecular structure inset. Protonation to form pentylammonium leads to a pronounced increase in positive charge localized on the ammonium group. Here, we have reservations about the applicability of the CHELPG charges. The carbon of CH_2^a has a charge of +0.60, compared to +0.29 for the corresponding carbon in fluoropentane. This is certainly not what we would expect. Nor would we expect an NH_2 group to be more electron-withdrawing from this position than an NH_3^+ group. However, in line with the other charge models, there is a larger electron-withdrawing effect from C5 than from C4 or C3.

***Anti* Vicinal NBO Interactions (kcal mol⁻¹) in Pentylamine and Pentylammonium**

Table S8: Natural bond orbital (NBO) second-order perturbation stabilization energies (in kcal mol^{-1}) for donor–acceptor interactions in Pentylamine and Pentylammonium with no applied field. Hyperconjugation interactions involving the N–H bonds or the nitrogen lone pair are not shown, since they cannot be directly compared across the two compounds. A number of geminal NBO interactions are present in the pentylammonium cation. These have been omitted for clarity.

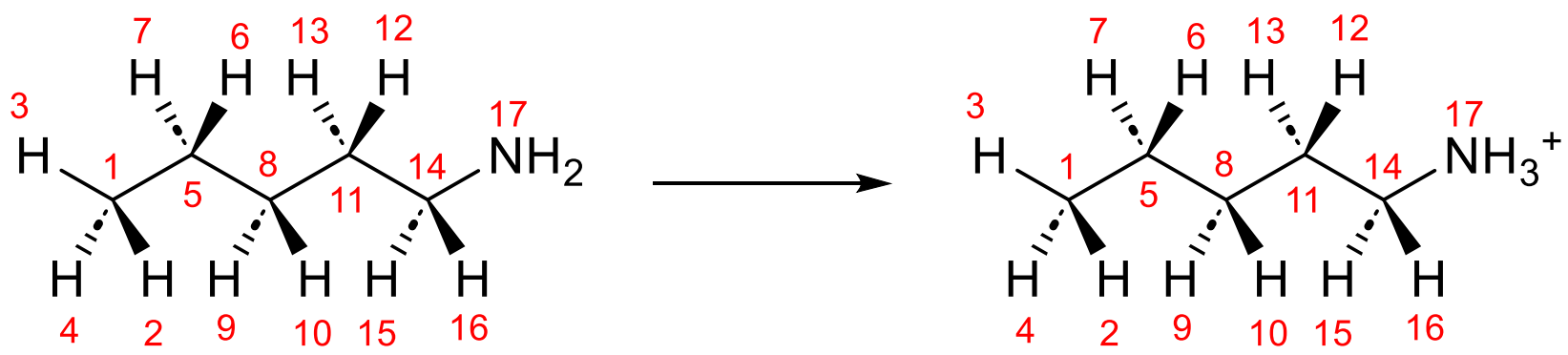

Donor (σ)	Acceptor (σ*)	Amine	Ammonium	Difference
C1–H2	C5–H7	3.73	3.82	+0.09
C1–H3	C5–C8	3.62	4.00	+0.38
C1–H4	C5–H6	3.73	3.82	+0.09
C1–C5	C8–C11	2.62	3.04	+0.42
C5–H6	C1–H4	3.75	3.44	–0.31
C5–H6	C8–H10	4.19	4.27	+0.08
C5–H7	C1–H2	3.75	3.44	–0.31
C5–H7	C8–H9	4.19	4.27	+0.08
C5–C8	C1–H3	1.97	1.73	–0.24
C5–C8	C11–C14	2.30	2.79	+0.49
C8–H9	C5–H7	3.90	3.46	–0.44
C8–H9	C11–H12	4.17	4.22	+0.05
C8–H10	C5–H6	3.90	3.46	–0.44
C8–H10	C11–H13	4.17	4.22	+0.05
C8–C11	C1–C5	2.57	2.21	–0.36
C8–C11	C14–N17	3.18	4.58	+1.40
C11–H12	C8–H9	4.06	3.37	–0.69
C11–H12	C14–H15	3.72	4.04	+0.32
C11–H13	C8–H10	4.06	3.37	–0.69
C11–H13	C14–H16	3.72	4.04	+0.32
C11–C14	C5–C8	2.39	1.95	–0.44
C14–H15	C11–H12	4.10	3.26	–0.84
C14–H16	C11–H13	4.10	3.26	–0.84
C14–N17	C8–C11	1.68	1.47	–0.21

tert-Butyl and 2-Methylhex-2-yl Carbocations

Table S9: NBO second-order perturbation stabilization energies (kcal mol^{-1}) for σ(C–H) → p(C$^+$) hyperconjugative interactions stabilizing the *tert*-butyl carbocation. Strong donation is observed from one C–H bond on each methyl group, with weaker contributions from the remaining C–H bonds.

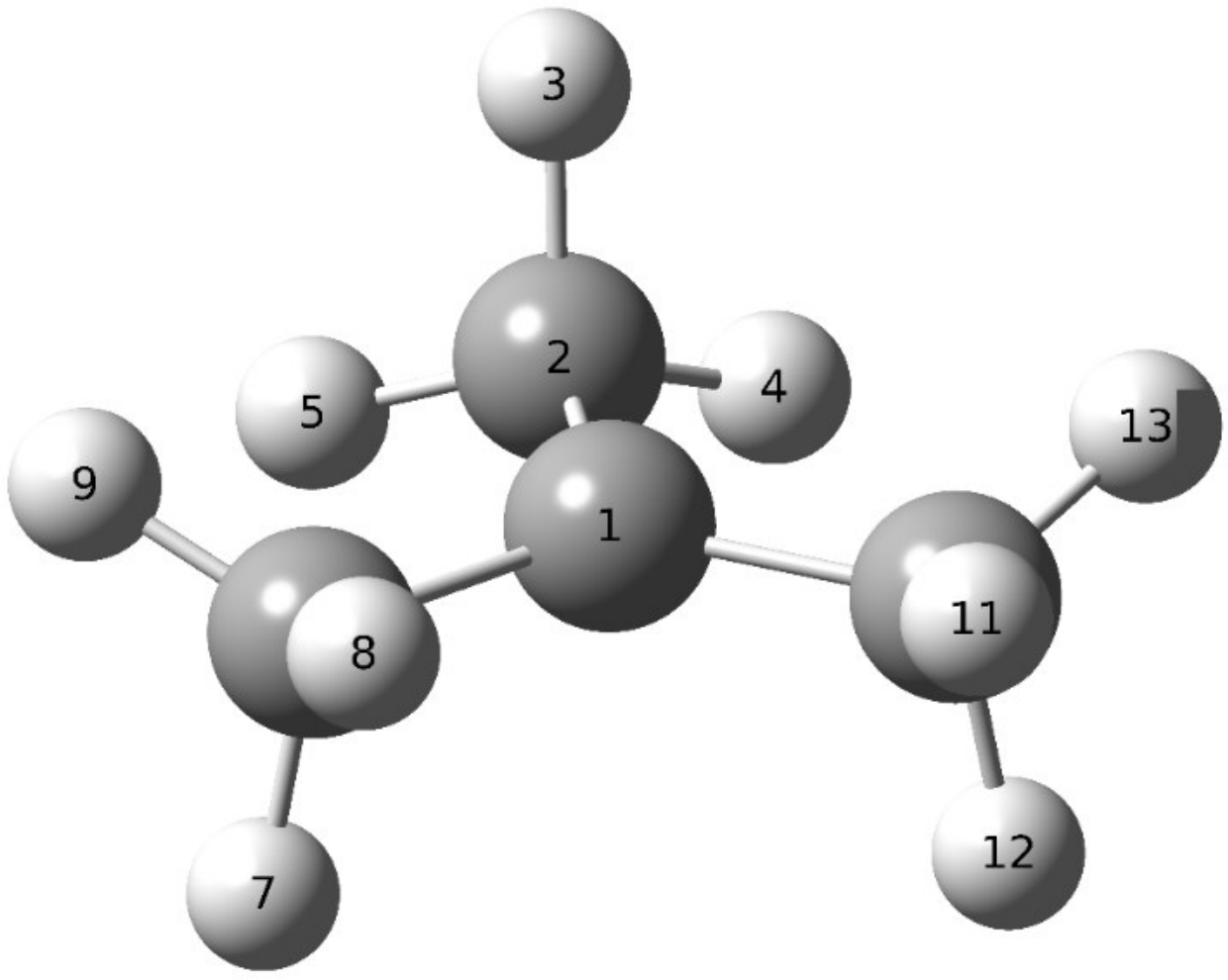

Donor (σ)	Acceptor (p)	Energy (kcal mol^{-1})
C2–H3	C1	35.47
C2–H4	C1	2.97
C2–H5	C1	2.95
C6–H7	C1	32.46
C6–H8	C1	2.43
C6–H9	C1	4.43
C10–H11	C1	2.46
C10–H12	C1	32.46
C10–H13	C1	4.39

Table S10: NBO second-order perturbation stabilization energies (kcal mol^{-1}) for σ(C–H) → p(C^+) hyperconjugative interactions in the 2-methylhex-2-yl carbocation. Overall stabilization is slightly smaller than in the tert-butyl carbocation. The C6–C9 and C19–H22 bonds are nearly orthogonal to the carbocation p-orbital and therefore provide negligible stabilization.

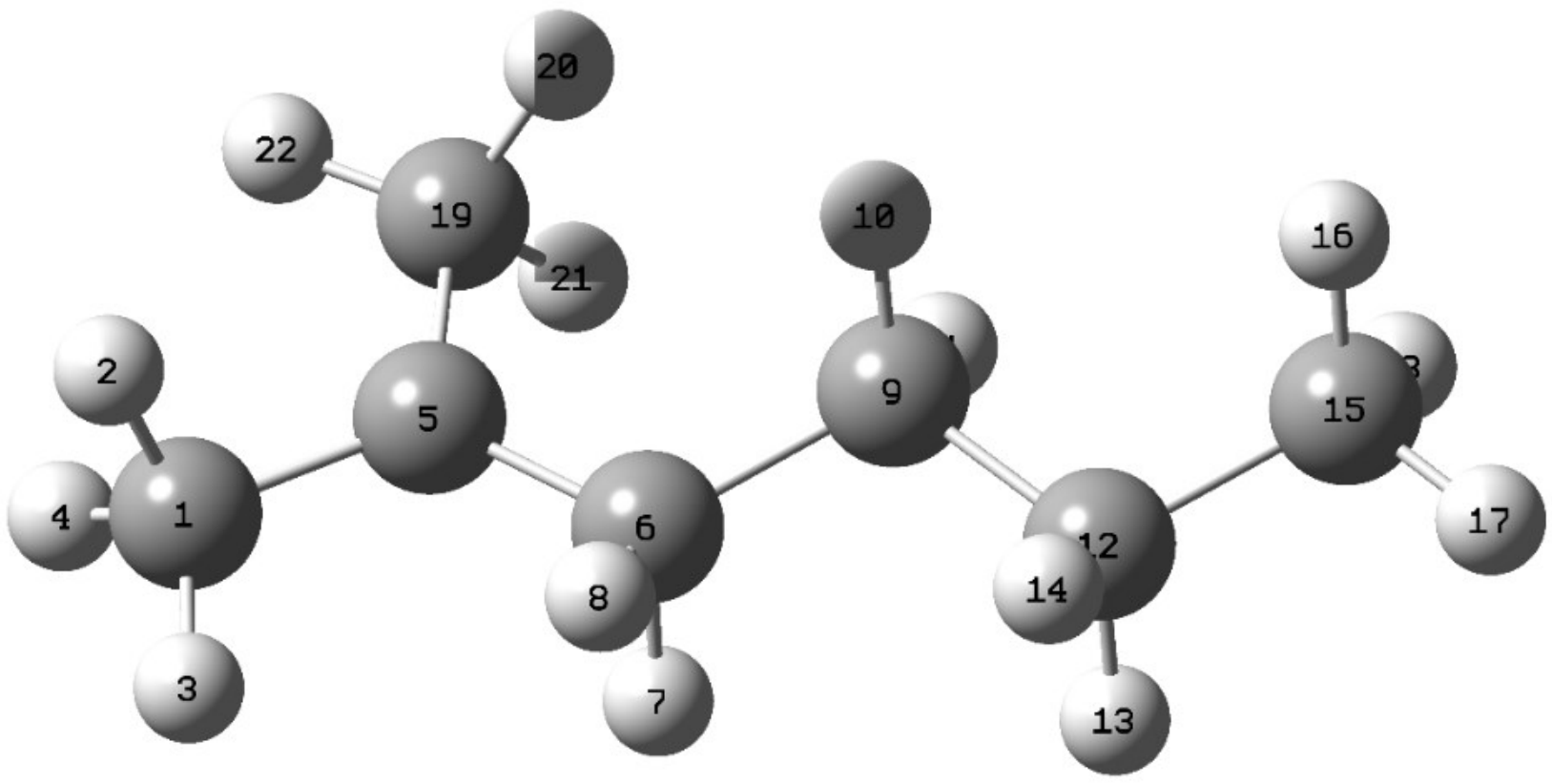

Donor (σ)	Acceptor (p)	(kcal mol^{-1})
C1–H2	C5	30.03
C1–H3	C5	1.02
C1–H4	C5	6.60
C6–H7	C5	37.56
C6–H8	C5	7.12
C19–H20	C5	14.97
C19–H21	C5	19.37

Table S11: NBO second-order perturbation stabilization energies (kcal mol^{-1}) for *anti* vicinal σ(C–H) → σ(C–H) interactions along the butyl chain of 2-methylhexane (alkane) and the corresponding 2-methylhex-2-yl carbocation. Interactions oriented away from the carbocation center decrease in strength, whereas those directed toward the carbocation increase, consistent with the enhanced electron-withdrawing character of the cationic site.

Donor (σ)	Acceptor (σ*)	Alkane (kcal mol^{-1})	Cation (kcal mol^{-1})	Difference (kcal mol^{-1})
C6–H7	C9–H10	4.23	3.46	–0.77
C6–H8	C9–H11	4.14	2.91	–1.23
C9–H10	C12–H13	3.94	3.31	–0.63
C9–H11	C12–H14	3.92	3.27	–0.65
C12–H13	C15–H16	3.76	3.35	–0.41
C12–H14	C15–H18	3.77	3.37	–0.40
C15–H16	C12–H13	3.72	3.85	0.13
C15–H18	C12–H14	3.72	3.82	0.10
C12–H13	C9–H10	4.08	4.30	0.22
C12–H14	C9–H11	4.23	4.23	0.00
C9–H10	C6–H7	3.91	4.63	0.72
C9–H11	C6–H8	4.31	4.63	0.32

The following series of diagrams correspond to **Figure 17** in the manuscript, with other charge models.

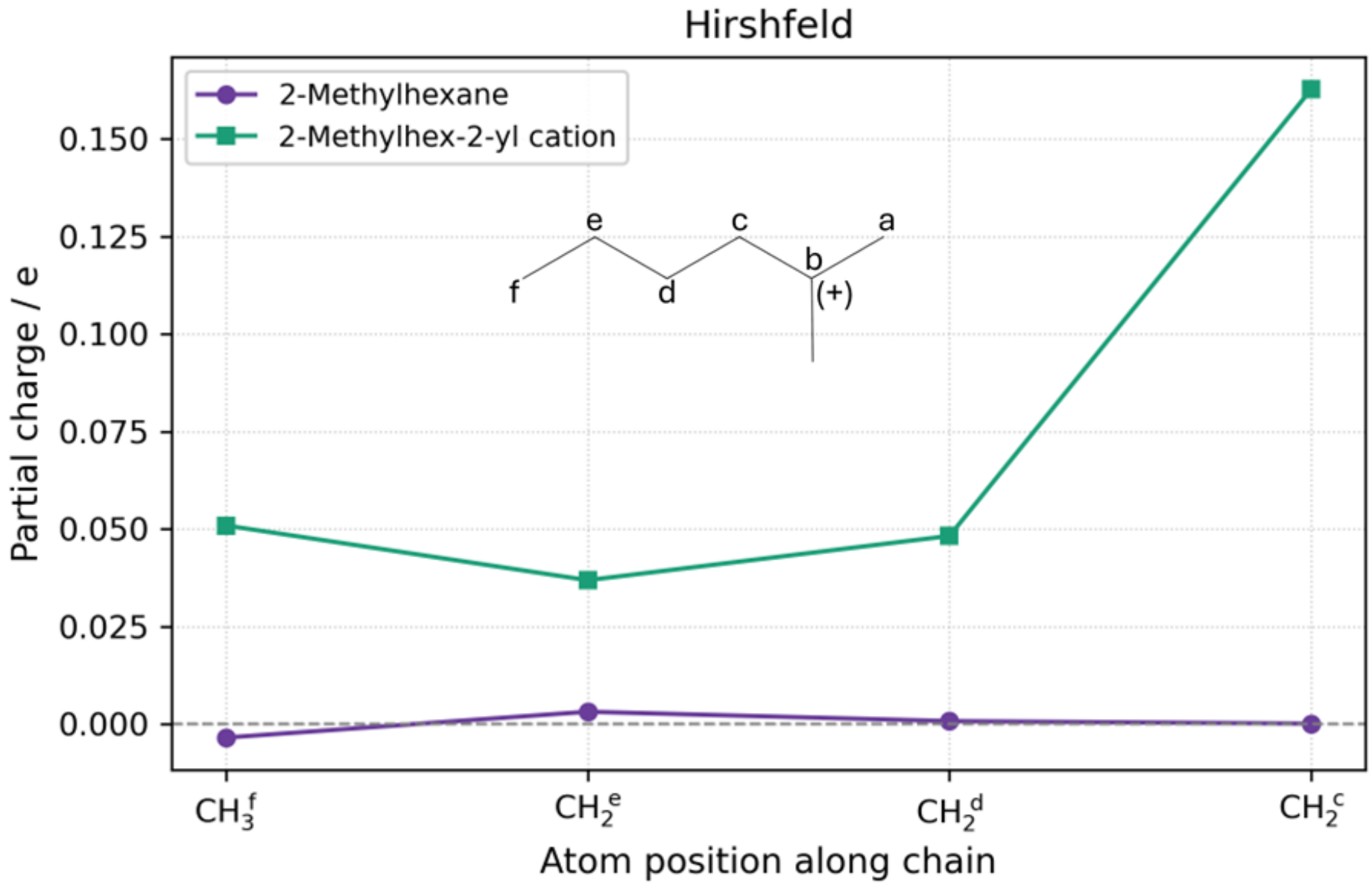

Figure S15. Calculated Hirshfeld charges in the butyl group in the 2-methylhex-2-yl carbocation and in 2-methylhexane.

NPA charges appear somewhat strange, although there are parallels. The key difference is that there appears to be net electron-donation to the CH_2 attached to the carbocation. However, this represents a significant electron-withdrawing effect (polarization/hyperconjugation) balanced by donation of electron-density to C. We note that others have seen a similar effect.[12]

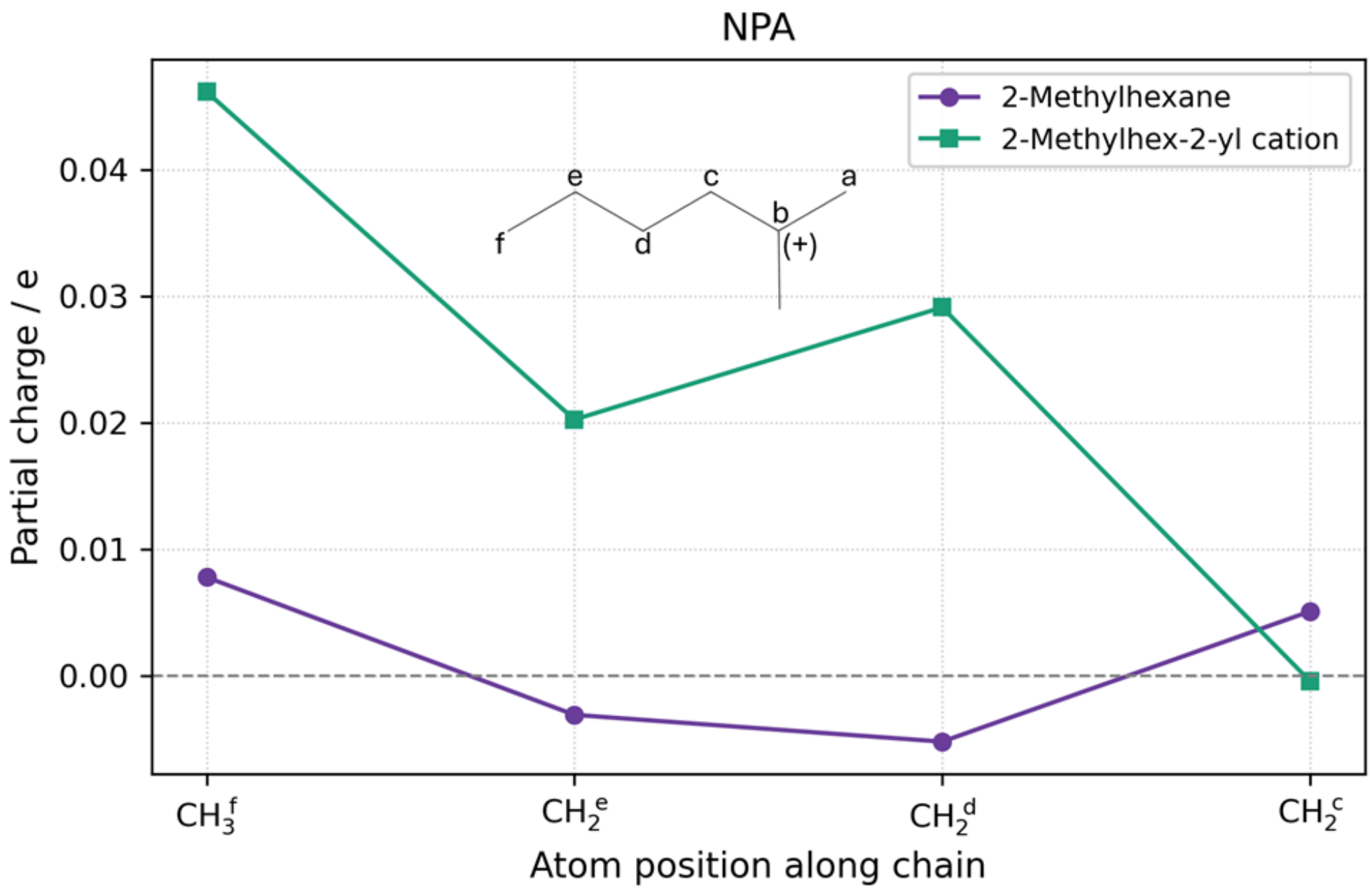

Figure S16. Calculated NPA charges in the butyl group in the 2-methylhex-2-yl carbocation and in 2-methylhexane.

Changes in CHELPG charges are broadly in line with those in CM5 charges, although the actual charges look unusual as already discussed. The same is true for DDEC6 charges.

[12] K. B. Wiberg, P. von Rague Schleyer and A. Streitweiser, *Can. J. Chem.* 1996, **74**, 892–900.

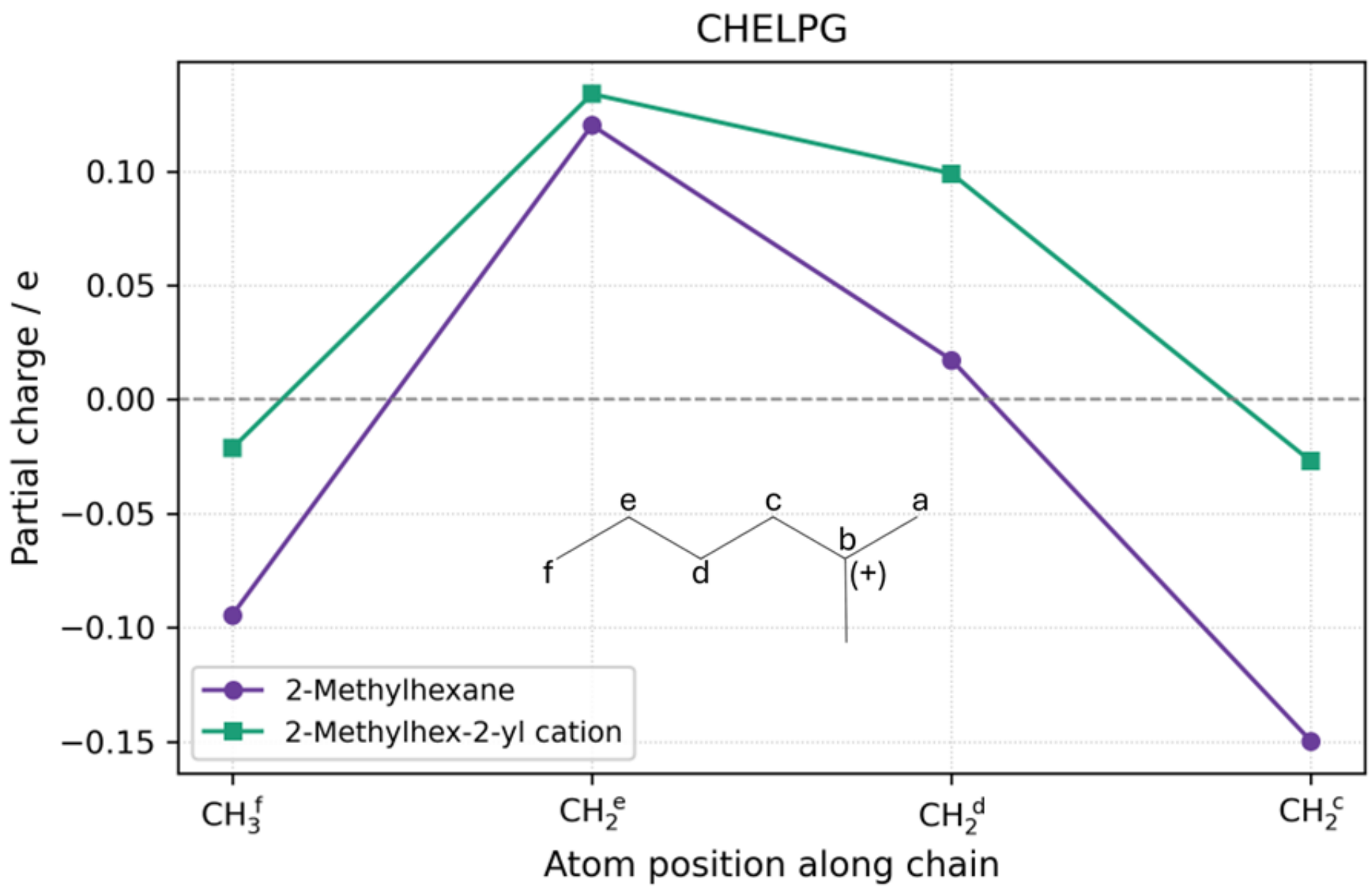

Figure S17. Calculated CHELPG charges in the butyl group in the 2-methylhex-2-yl carbocation and in 2-methylhexane.

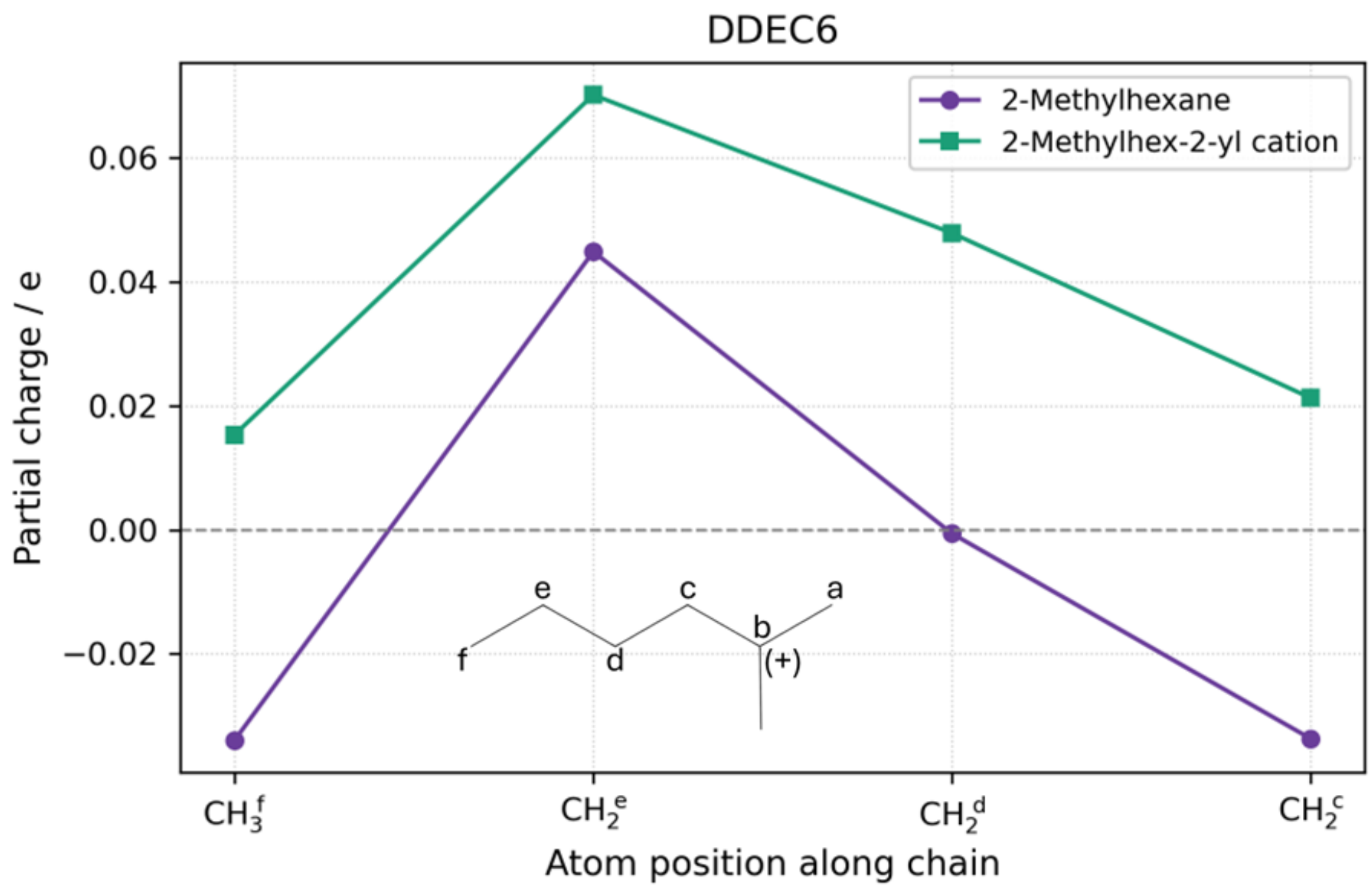

Figure S18. Calculated DDEC6 charges in the butyl group in the 2-methylhex-2-yl carbocation and in 2-methylhexane.

This is seen more clearly if we examine the change in charge between the alkane and the carbocation (**Figure S19**). In all charge models, there is more electron-withdrawal from the terminal CH_3 than from its adjacent CH_2 group. The effects at the point of attachment (right) are generally higher, with the exception of the NPA charges as discussed above.

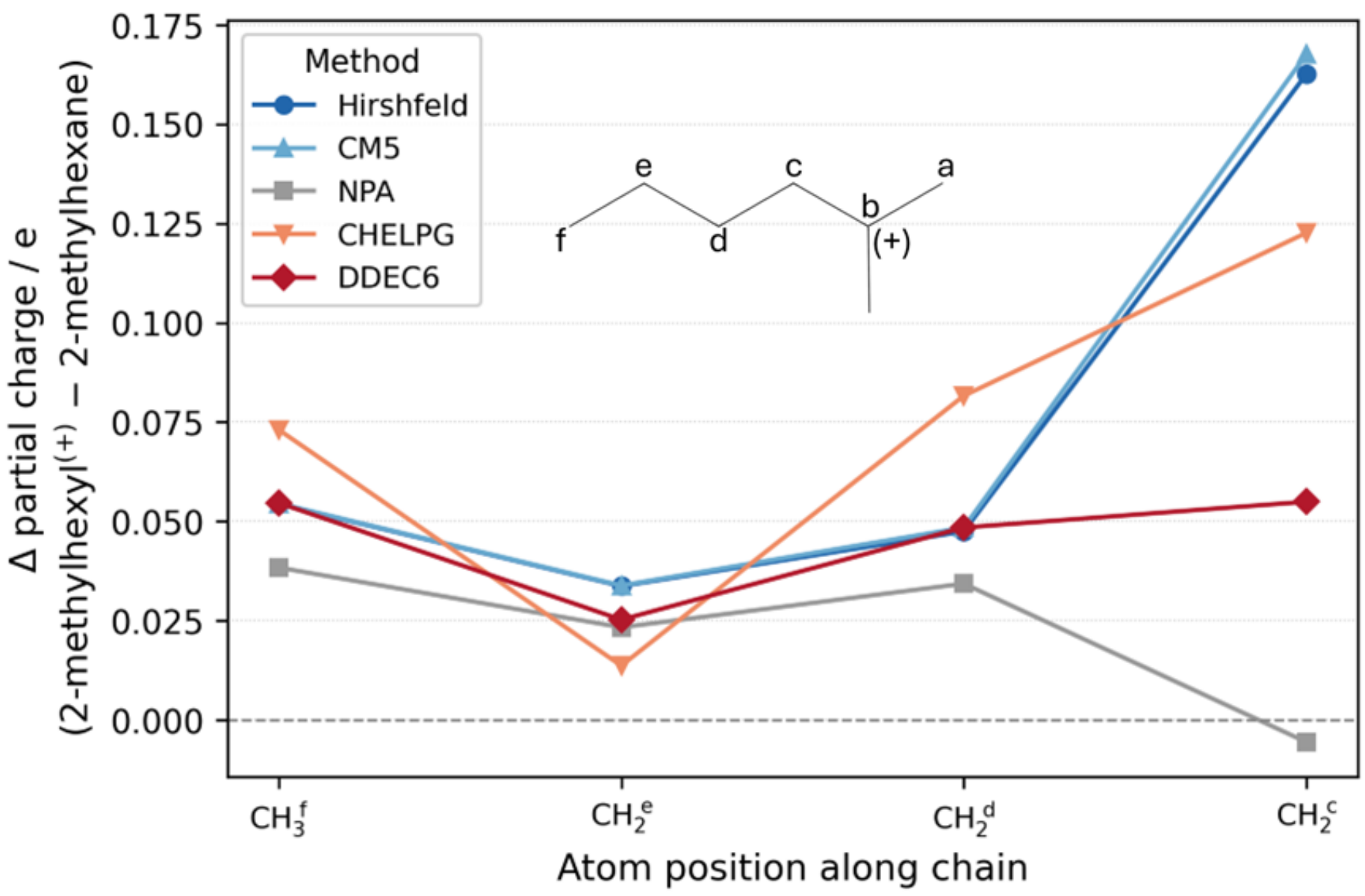

Figure S19. Partial charges of groups along the butyl substituent of the 2-methylhex-2-yl carbocation relative to 2-methylhexane, calculated using different charge decomposition methods (Hirshfeld, CM5, NPA, CHELPG, DDEC6).

The computational files are available via the Cardiff University Data Catalogue as described in the manuscript.
